# Effects of humic electron mediators on reductive dechlorination of polychlorinated biphenyl by consortia enriched from terrestrial and marine environments

**DOI:** 10.3389/fmicb.2024.1452787

**Published:** 2024-08-01

**Authors:** Qiong Wang, Dongdong Zhang, Xinkai Li, Yi Wang, Heng Wang, Zhichao Zhang, Wei Song, Peng Guo

**Affiliations:** ^1^Institute of Agricultural Products Processing and Nuclear Agriculture Technology Research, Hubei Academy of Agricultural Sciences, Wuhan, China; ^2^School of International Studies, Ningbo University, Ningbo, China; ^3^Donghai Laboratory, Zhoushan, China; ^4^Institute of Marine Biology and Pharmacology, Ocean College, Zhejiang University, Zhoushan, China; ^5^Key Laboratory of Health Risk Factors for Seafood of Zhejiang Province, Zhoushan, China; ^6^Hubei Hongshan Laboratory, Wuhan, China

**Keywords:** PCBs, humic electron mediator, microbial reductive dichlorination, terrestrial and marine consortia, *Dehalococcoides*

## Abstract

Humic electron mediators can facilitate the reductive dehalogenation of organohalogenated compounds by accelerating electron transfer. To investigate the effect of humic electron mediators on the microbial anaerobic reductive dechlorination of Polychlorinated biphenyls (PCBs), three types of humic electron mediators, humin (HM), humic acid (HA), and anthraquinone-2,6-disulfonic acid (AQDS, HA analogs), were added to PCB dechlorination cultures enriched from different sources in terrestrial and marine environments (T and M cultures). The results showed that meta- and para-site dechlorination occurred in the M culture, while only meta-site dechlorination occurred in the T culture. The dechlorination process N and the dechlorination process H or H′ are presented in both cultures. HM enhanced PCB dechlorination metabolic activity in both cultures mainly by promoting meta-site dechlorination. HA showed a weak promoting effect on the M culture by promoting para-chlorine removal but inhibited the dechlorination metabolism of the terrestrial-origin culture, inhibiting meta-chlorine removal. AQDS showed inhibitory effects on both cultures by inhibiting the microbial removal of meta-chlorine. High-throughput sequencing and qPCR results suggest that HM is not a carbon source for the potential dechlorinating metabolism of *Dehalococcoides* but may promote reductive dechlorination by changing the community structure, and AQDS may inhibit anaerobic reductive dechlorination of PCBs by inhibiting the growth of *Dehalococcoides*. This study provides insights into the mechanism of enhancing PCB microbial dechlorination mediated by humic substances and plays a significant role in extending the application prospects of PCBs bioremediation technology.

## 1 Introduction

Bioremediation methods for polychlorinated biphenyl (PCB) contamination have gained significant attention owing to their potential as environmentally friendly and economically sustainable approaches. Sediments, which are crucial reservoirs of PCBs in the environment, may harbor anaerobic dechlorinating bacteria capable of degrading PCBs. However, current research on the anaerobic dechlorination of PCBs in sediments has primarily focused on terrestrial and freshwater environments (Wiegel and Wu, 2000; Hughes et al., 2010), with limited studies on PCB dechlorination in marine sediment environments. This unique marine environment may harbor novel dehalogenating microorganisms and enzymes (Zanaroli et al., 2015), offering potential new microbial resources for PCB biodegradation. Given that PCBs have historically been used as commercial mixtures, such as Aroclor 1260, they are mostly present in the environment as mixtures rather than as single congeners, rendering them more toxic, less bioavailable, and consequently more difficult to degrade (Bedard et al., 2007; Fagervold et al., 2007).

Humic substances, categorized based on solubility, include fulvic acid (soluble at all pH conditions), humic acid (insoluble at pH < 2 and soluble at higher pH), and humin (insoluble at all pH conditions), are electron mediators in microbial redox reactions (Van der Zee and Cervantes, 2009; Zhang and Katayama, 2012; Zhang et al., 2014; Lipczynska-Kochany, 2018). Humic electron mediators can accelerate interspecies electron transfer, thereby promoting the microbial reductive dehalogenation of organohalides (Zhang and Katayama, 2012; Zhang et al., 2014). Previously, research on humic substances largely focused on soluble fractions (Pham et al., 2021), with little attention given to the impact of solid-phase redox mediator on microbial activity, despite its potentially significant role in microbial reductive dehalogenation (Guo et al., 2018). However, comparative analyses of the impact of humic substance electron mediators on the dechlorination rates of PCB-dechlorinating cultures enriched from terrestrial and marine sediment environments are limited. This unique marine environment may shape distinct microbial communities in marine sediments, suggesting that the influence of humic electron mediators on PCB-dechlorinating cultures originating from marine environments may differ from those sourced from terrestrial environments.

To investigate the effects of humic substance as electron mediators on anaerobic dechlorination by cultures originating from different environments, humin (HM), humic acid (HA), and anthraquinone-2,6-disulfonic acid (AQDS, HA analogs), were added to PCB-dechlorinating cultures enriched from terrestrial and marine environments. The reductive dechlorination activity of the commercial PCB mixture, Aroclor 1260, was assessed in various treatment groups. Additionally, high-throughput sequencing and qPCR were used to explore the different influences of various humic substances as electron mediators on the microbial community structure, growth of functional bacteria, and interspecies metabolic interactions in dechlorinating cultures enriched from terrestrial and marine sources.

## 2 Materials and methods

### 2.1 Humic substance electron mediators mediate the metabolism of PCB dechlorinating cultures from different sources

To investigate the influence of humic substance electron mediators on the metabolism of PCB-dechlorinating cultures from terrestrial and marine sources, three different experimental treatment groups were separately added to the culture systems of PCB-dechlorinating cultures enriched from terrestrial and marine sources.

#### 2.1.1 Humic substance electron mediators

HA was purchased from Sigma-Aldrich (Shanghai), and AQDS was obtained from TCI Development Co. Ltd. (Shanghai, China). HM cannot be purchased commercially as a standard product and must be extracted from sediment samples. The specific extraction steps are as follows: After air-drying sediment samples, they were stored at 4°C. Approximately 1.5 kg of sediment was weighed and placed in a 250 mL centrifuge bottle. Then, 150 mL of 0.1 M sodium hydroxide solution was added to the centrifuge bottle, which was then placed on a shaker for 24 h (170 rpm, 30°C, same below). After shaking, the mixture was centrifuged (5,000 rpm, 15 min) and the supernatant was discarded to complete the alkaline extraction. Alkaline extraction was repeated at least four times until the supernatant became light tea–colored. After centrifugation, the precipitate was treated with 2% hydrofluoric acid for acid extraction. The acid extraction was repeated twice, and the precipitate was washed with water until neutral. Finally, the precipitate, which constituted the HM, was collected after centrifugation. The HM used in this experiment was extracted from sediment samples collected from fishponds in Huzhou (30°46′38″, 120°9′5″), as reported in previous studies (Zhang and Katayama, 2012; Zhang et al., 2017). The physicochemical characteristics are presented in Supplementary Table S1.

#### 2.1.2 Preparation of anaerobic culture medium

An anaerobic culture medium was prepared according to the method described by Zhang et al. (2014). Prior to preparation of the medium, various stock solutions were prepared. The formulae for the stock solutions used in this experiment are shown in Supplementary Tables S2–S6. The components of the anaerobic medium before sterilization are listed in Supplementary Table S7.

HA stock solution was prepared by dissolving 0.375 g of HA in 40 mL of 0.1 M NaOH solution. The solution was stirred until completely dissolved and the pH was slowly adjusted to 7 using dilute hydrochloric acid. Finally, the volume was adjusted to 50 mL. The HA concentration was 7.5 g/L. Remove oxygen from the solution by purging it with nitrogen gas and store at 4°C. The volume of the solution was ensured not to exceed 50 mL during pH adjustment.

AQDS stock solution was prepared by dissolving 0.4947 g of AQDS in 50 mL of ultrapure water. Oxygen was removed by purging with nitrogen gas and stored at 4°C. The concentration of the AQDS stock solution was 24 mM. The specific preparation method for the anaerobic culture medium is described in the Supplementary materials and methods.

#### 2.1.3 Microbial sources

Sediments are important sinks for PCBs. Two dechlorinating microbial cultures, referred to as the M and T cultures, were enriched in sediments collected from the East China Sea and a land rice paddy field. The M culture was enriched from sediments near the coast of the East China Sea (29°18′95″N, 122°22′48″E), sampled near the coast of Taizhou. During the cruise of Dongfanghong 2 in April 2017, sediment was collected using a stainless-steel Gray O’Hara box corer, and undisturbed sediment from the surface layer (0–5 cm) was immediately transferred to serum bottles containing anaerobic culture medium (1:1 volume ratio). The bottles were sealed with rubber stoppers and aluminum foil, and the headspace air was replaced with high-purity nitrogen gas before the enrichment culture. The T culture was enriched from sediment in a rice paddy field in Taizhou (28°54′84″N, 121°37′33″E), provided by the research group of Shen Chaofeng from the College of Environmental and Resource Sciences, Zhejiang University. Taizhou is one of the largest electronic waste discharge and treatment centers in China. Cultures from different terrestrial and marine sources were enriched around Taizhou City, with the two enrichment sites located <86 km apart (Supplementary Figure S1), to minimize the influence of spatial environmental variables on the cultures.

#### 2.1.4 Experimental design

In this study, three different experimental groups were established by separately adding HM, HA, or AQDS to enriched M and T cultures. Biotic control group consisting of a dechlorination culture system without the addition of electron mediators was included. The final concentrations of HM, HA, and AQDS in the culture systems were approximately 6.67 g/L, 0.125 g/L, and 1 mM, respectively. In this study, M-Bla and T-Bla represent the M and T culture systems, respectively, without the addition of any electron mediators. M-HM and T-HM represent the M and T culture systems with the addition of HM, respectively. M-HA and T-HA represent the M and T culture systems with HA, respectively. M-AQDS and T-AQDS represent M and T culture systems containing AQDS, respectively. Each treatment group included a corresponding abiotic control group without microbial inoculation, and three parallel culture systems were established for each of the control and experimental groups.

#### 2.1.5 Addition of PCBs

Fifty milligrams of Aroclor 1260 (Accustandard, New Haven, CT, United States) were dissolved in 2 mL of acetone by vortexing to prepare a 25 g/L Aroclor 1260 stock solution, which was stored in a 4°C refrigerator. Thirty microliters of the stock solution were added to 30 mL of anaerobic culture medium using a microliter syringe, resulting in a final concentration of Aroclor 1260 of 25 mg/L Aroclor 1260.

#### 2.1.6 Extraction and analysis of PCBs

The mother liquors (M and T culture systems showing PCB dechlorination activity) were shaken in an anaerobic chamber. Two milliliters of the mother liquor were inoculated into serum bottles containing anaerobic culture medium using a disposable syringe, establishing dechlorinating microbial cultures for PCBs. Cultures were maintained at 30°C in an anaerobic workstation. During cultivation, periodic sampling was conducted to monitor the PCB dechlorination activity of the microbial cultures. The PCB extraction method for PCBs was described in the Supplementary materials and methods. PCBs were qualitatively and quantitatively analyzed using gas chromatography–mass spectrometry GC–MS (QP2020, Shimadzu, Kyoto, Japan). The temperature program is presented in Supplementary Table S8. Qualitative analysis of each PCB congener in the samples was completed by comparing the retention times and integrated ion signals of Aroclor 1260 and its metabolites with a mixture of nine PCBs congener standards (Accustandard, New Haven, CT, United States, containing 209 congeners). The molar percentages of PCB congeners with different chlorine contents were used to calculate the average number of chlorine atoms per PCB molecule in the culture system. PCB dechlorination rates in the culture system were calculated based on the average number of chlorine atoms per PCB molecule.

### 2.2 Analysis of PCB dechlorinating microbial community

#### 2.2.1 Sample collection and DNA extraction

Samples were collected when the microbial cultures in the serum bottles showed significant PCB degradation activity. Because M-Bla, M-HM, and M-HA exhibited significant dechlorination activity on day 21, samples from these three treatment groups were selected for high-throughput sequencing on day 21. In contrast, M-AQDS exhibited significant dechlorination activity only on day 112; therefore, samples from day 112 were chosen for high-throughput sequencing of M-AQDS. Similarly, T-Bla, T-HM, and T-HA samples from day 21 were selected for high-throughput sequencing, whereas those from day 45 were chosen for high-throughput sequencing of T-AQDS. The culture in the serum bottles was thoroughly mixed, and 3 mL of culture was transferred into two 2 mL centrifuge tubes and immediately stored at −80°C. Due to the presence of humic substances in some samples, DNA was extracted from the samples using the E.Z.N.A.^®^ Soil DNA kit (OMEGA, United States) according to the manufacturer’s instructions. The concentration of the extracted DNA was measured using a NanoDrop spectrophotometer (NC2000; Thermo Scientific).

#### 2.2.2 16S rRNA gene high-throughput sequencing

High-throughput sequencing services were provided by Shanghai Personal Biotechnology Co. Ltd. The V3-V4 region of bacterial 16S rRNA was amplified by universal primers 338F (5′-ACTCCTACGGGAGGCAGCA-3′) and 806R (5′-GGACTACHVGGGTWTCTAAT-3′) (Eisen et al., 2008; Caporaso et al., 2010). Bioinformatic analysis was conducted using QIIME 2 (Version 2019.4) (Bokulich et al., 2018). The DADA2 method was used for primer removal, quality filtering, denoising, merging, and chimera removal to obtain amplicon sequence variants and feature tables (Callahan et al., 2016). Three replicates were analyzed for each treatment group.

#### 2.2.3 Real-time quantitative PCR and phylogenetic tree construction

PCR amplification of target genes from extracted DNA was performed using specific primers to obtain target gene fragments. Specific primers were used to amplify the 16S rRNA genes of *Dehalococcoides*, *Dehalogenimonas*, *Dehalobacter*, and *Dehalobium* as well as the full-length *Dehalococcoides* 16S rRNA gene. Specific primers were also used to amplify the PCB-degrading functional genes (*pcbA1*, *pcbA4*, *pcbA5*) of *Dehalococcoides*, as well as the full-length reductive dehalogenase gene. The primer sequences are listed in Supplementary Table S9. The PCR and qPCR procedures are listed in Supplementary Tables S10, S11, respectively. PCR and qPCR were conducted as described in Supplementary materials and methods. MEGA software (version 7.0) (Kumar et al., 2016) was used to construct phylogenetic trees based on the neighbor-joining method, with the bootstrap set to 1,000 replicates.

#### 2.2.4 Statistical analysis

Tukey’s honest significant difference (HSD) test was performed to analyze the differences between the treatment groups. Non-metric multidimensional scaling (NMDS), permutational multivariate analysis of variance (Adonis), and analysis of similarity (ANOSIM)were conducted to compare the bacterial community composition between different groups. To evaluate the interactions between microbial communities at the genus level, Spearman’s correlation coefficients were calculated using the *psych* package in R 4.2.2 (Ihaka and Gentleman, 1996) for genera with relative abundances greater than 0.01%. Co-occurrence network analyses were performed separately for M and T cultures using the interactive platform Gephi v.0.9.2 (Bastian et al., 2009). Only statistically significant (*p* < 0.05) correlations with an absolute correlation coefficient greater than 0.7 (|r| > 0.7) were analyzed. Linear discriminant analysis effect size (LEfSe) analysis was performed at the genus level using MicrobiomeAnalyst^1^ (Chong et al., 2020), the Kruskal–Wallis rank sum test, and linear discriminant analysis.

## 3 Results

### 3.1 Influence of humic substances electron mediators on PCBs reductive dechlorination activity of microbial communities

#### 3.1.1 Influence of humic substances electron mediators on marine-origin PCBs reductive dechlorination activity of microbial communities

**Figure 1:**
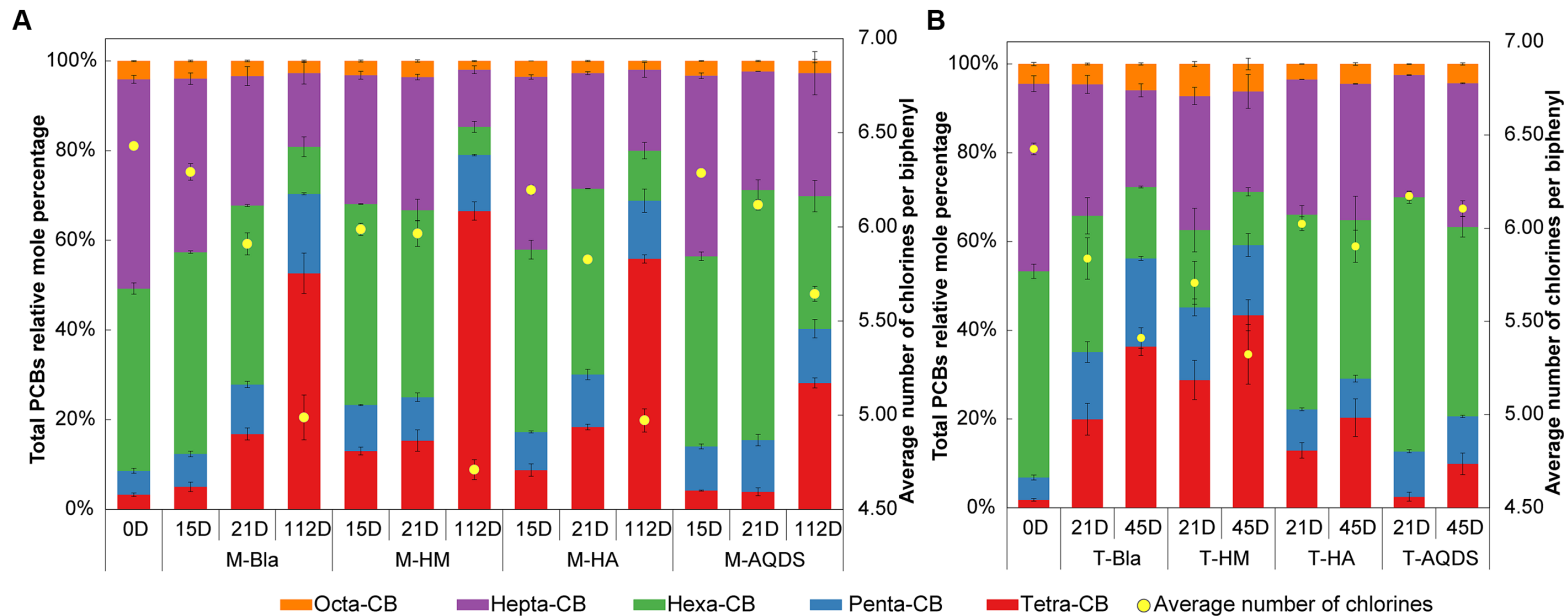
Mole percentages of polychlorinated biphenyls (PCBs) with different chlorination degrees and the average number of chlorines per biphenyl in various culture systems enriched from the marine **(A)** and terrestrial **(B)** sediment, respectively. M and T represent the PCB dechlorination cultures enriched from terrestrial and marine environments, respectively. HM, HA, and AQDS represent the culture systems with humin, humic acid, and anthraquinone-2,6-disulfonic acid, respectively. Bla represents the culture systems without the addition of any electron mediators.

The effect of humic substances electron mediators on the reductive dechlorination activity of the M microbial community is shown in Figure 1A. In this experiment, no dichlorobiphenyls or trichlorobiphenyls were detected, and tetrachlorobiphenyls were the final dechlorination metabolites in all M microbial community cultures. Supplementary Figure S2A shows that, at the end of the cultivation experiment (112 d later), no dechlorination activity was detected in the abiotic controls. M-HM showed significant dechlorination activity on day 15. In the M-HM system, the average chlorine atom number per PCB molecule decreased from 6.34 on day 0 to 5.99 on day 15, with a dechlorination rate of 1.98 μM Cl^−^ d^−1^. On day 15, the average numbers of chlorine atoms per PCB molecule in the M-Bla, M-HA, and M-AQDS systems were 6.29, 6.20, and 6.29, respectively, with dechlorination rates of 0.62, 1.05, and 0.64 μM Cl^−^ d^−1^.

On Day 21, both M-Bla and M-HA exhibited significant degradation. In the M-Bla and M-HA systems, the average chlorine atom numbers per PCB molecule decreased to 5.91 and 5.83, respectively, with average dechlorination rates of 4.28 and 4.14 μM Cl^−^ d^−1^ between day 15 and day 21. The average dechlorination rates for the first 21 days were 1.66 and 1.93 μM Cl^−^ d^−1^, respectively. In contrast, the M-HM system exhibited lower degradation rates than the M and M-HA systems between days 15 and 20, with degradation rates of only 0.25 μM Cl^−^ d^−1^. On day 21, the average chlorine atom number per PCB molecule in the M-HM system was 5.97, with an average dechlorination rate of 1.49 μM Cl^−^ d^−1^ for the first 21 days. Meanwhile, M-AQDS still showed weak degradation activity on day 21, with the average chlorine atom number per PCB molecule in the M-AQDS system being 6.12, and the average dechlorination rate between day 15 and day 21 being only 1.89 μM Cl^−^ d^−1^, with average dechlorination rates for the first 21 days being 0.64 μM Cl^−^ d^−1^.

It was not until Day 112 that M-AQDS showed significant dechlorination activity. In the M-AQDS system, the average chlorine atom number per PCB molecule decreased to 5.64, with an average dechlorination rate of 0.47 μM Cl^−^ d^−1^ from day 0 to day 112. In contrast, in the M-Bla, M-HM, and M-HA systems, the average chlorine atom numbers per PCB molecule had already decreased to 4.99, 4.71, and 4.97, respectively, with average dechlorination rates of 0.87, 1.49, and 0.87 μM Cl^−^ d^−1^ for the first 112 days.

In the first 15 d, both HM and HA addition increased the PCB dechlorination rates of the M microbial community compared to M-Bla, with M-HM and M-HA having 3.19 and 1.69 times higher dechlorination rates, respectively. However, the addition of AQDS did not promote anaerobic reductive dechlorination by the M microbial community. In the first 15 d, the M-HM cultivation system exhibited the highest PCBs anaerobic reductive dechlorination activity.

From days 15 to 21, the dechlorination rate of M-HM decreased, entering a slow dechlorination phase with lower dechlorination activity than those of M-Bla and M-HA. At this time, the M-Bla and M-HA cultivation systems showed higher PCBs anaerobic reductive dechlorination activities, whereas M-AQDS still showed no significant dechlorination activity and remained in the lag phase. The rapid dechlorination of M-HM in the first 15 days led to the accumulation of significant amounts of tetrachlorobiphenyl and the intermediate pentachlorobiphenyl in the culture system. In the M-HM system, the sum of the molar percentages of tetrachlorobiphenyl and pentachlorobiphenyl increased rapidly from 8.59% on Day 0 to 23.29%. The significant accumulation of these dechlorination products reduced the anaerobic dechlorination rate of M-HM after 15 days. In contrast, on day 15, the sum of the molar percentages of tetrachlorobiphenyl and pentachlorobiphenyl in the M-Bla, M-HA, and M-AQDS systems were only 12.37, 17.24, and 14.04%, respectively.

On day 112, M-Bla, M-HM, and M-HA entered the slow degradation stage. During this phase, compared to M-Bla, M-HM still promoted anaerobic reductive dechlorination of the M microbial community. The average dechlorination rate of M-HM over 112 days was 1.66 times that of M-Bla, whereas the dechlorination rates of M-HA and M-Bla were equal. The average dechlorination rate of M-AQDS was only 75.81% that of M-Bla. Therefore, during the entire 112-day process of PCBs anaerobic reductive dechlorination, the presence of HM increased the reductive dechlorination activity of the cultivation system, showing the highest dechlorination activity among the treatment groups. The cultivation system exhibited efficient dechlorination activity after 15 days and then entered a slow degradation stage. HA showed higher dechlorination activity after 21 days, being 1.16 times that of M-Bla, whereas AQDS significantly inhibited anaerobic reductive dechlorination of the M microbial community.

#### 3.1.2 Influence of humic substances electron mediators on terrestrial-origin PCBs reductive dechlorination activity of microbial communities

The influence of electron mediators on the reductive dechlorination activity of the T microbial community is shown in Figure 1B. In this experiment, no dichlorobiphenyls or trichlorobiphenyls were detected, and tetrachlorobiphenyls were the final dechlorination metabolites in all T microbial community cultures. No dechlorination activity was detected in abiotic controls (Supplementary Figure S2B). No dechlorination activity was detected in either the different humic substance electron-mediator-treated groups or the control groups on day 15.

On day 21, except for T-AQDS, significant dechlorination activity was detected for T-Bla, T-HM, and T-HA. In the T-Bla, T-HM, and T-HA cultivation systems, the average chlorine atom numbers per PCB molecule decreased from day 0’s 6.4 to 5.8, 5.7, and 6.0, respectively, with dechlorination rates of 1.9, 2.3, and 1.3 μM Cl^−^ d^−1^, respectively. The sum of the molar percentages of tetrachlorobiphenyl and pentachlorobiphenyl in the system increased from day 0’s 6.8% to 35.1, 45.2, and 22.2%, respectively. However, in the T-AQDS system, the average number of chlorine atoms per PCB molecule was 6.2, with a dechlorination rate of only 0.8 μM Cl^−^ d^−1^. The sum of the molar percentages of tetrachlorobiphenyl and pentachlorobiphenyl in the T-AQDS system was 12.7%. On day 21, HM promoted the anaerobic reductive dechlorination of PCBs by the T microbial community, with a dechlorination rate 1.2 times higher than that of the control group. However, the presence of HA and AQDS inhibited the anaerobic reductive dechlorination activity of the T microbial community, especially AQDS, where the dechlorination rate of T-AQDS was only 43.1% of that of the biological control group.

After 45 d, significant dechlorination activity was detected in T-AQDS. The average chlorine atom number per PCB molecule in the T-AQDS system decreased to 6.1, with an average dechlorination rate of 0.5 μM Cl^−^ d^−1^ over 45 days. The sum of the molar percentages of tetrachlorobiphenyl and pentachlorobiphenyl increased to 20.6%, and the molar percentage of tetrachlorobiphenyl increased significantly from day 21 (2.5%) to 9.9% on day 45. After 45 days, the average number of chlorine atoms per PCB molecule in the T-Bla, T-HM, and T-HA systems decreased to 5.4, 5.3, and 5.9, respectively, with average dechlorination rates of 1.5, 1.7, and 0.8 μM Cl^−^ d^−1^. Throughout the 45-day dechlorination cultivation process, HM promoted the anaerobic reductive dechlorination activity of the T microbial community, whereas HA and AQDS exhibited certain inhibitory effects.

### 3.2 Inference of dechlorination metabolic pathways of PCBs microbial communities from different sources

**Figure 2:**
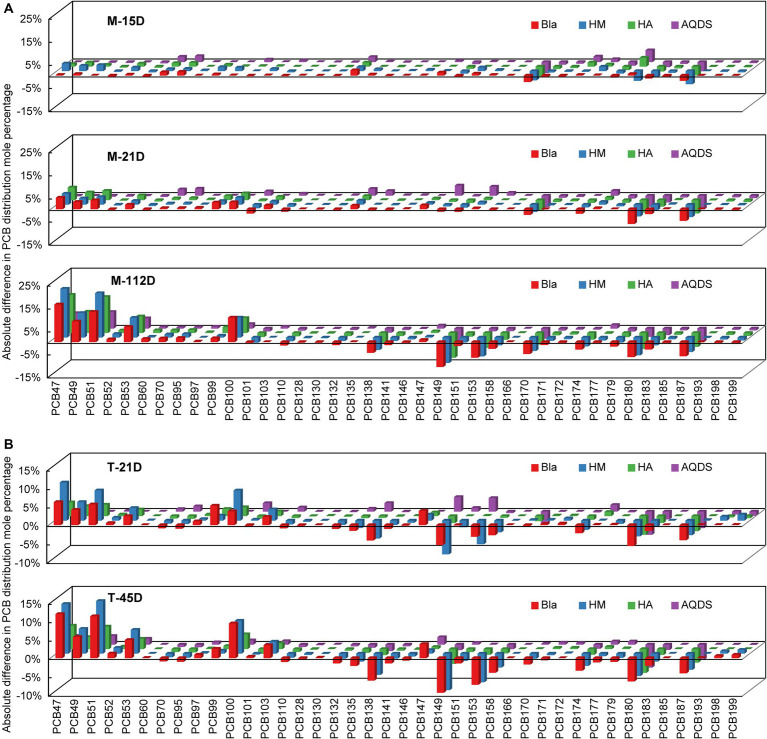
Reductive dechlorination of Aroclor 1,260 in various culture systems enriched from the marine sediment **(A)** on day15, day 21, and day 112, and the terrestrial sediment **(B)** on day 21 and day 45. M and T represent the PCB dechlorination cultures enriched from terrestrial and marine environments, respectively. HM, HA, and AQDS represent the culture systems with humin, humic acid, and anthraquinone-2,6-disulfonic acid, respectively. Bla represents the culture systems without the addition of any electron mediators.

Figure 2 shows the changes in the molar percentages of various PCB congeners over time in the M and T microbial communities. PCB47, 49, 51, and 53 were the main dechlorination end products in all the dechlorination systems. Compared with the composition of PCB congeners on day 0, the molar percentages of PCB138, 149, 153, 174, 180, and 187 decreased significantly. The main PCB dechlorination metabolites in the experimental groups treated with electron mediators were similar to those in the control group.

In microbial community system M, on day 0, the total molar percentages of PCB47, 49, 51, and 53 were only 0.4%. After 112 days, these four congeners increased to 47.0, 61.6, 50.8, and 25.2% in the M-Bla, M-HM, M-HA, and M-AQDS systems, respectively (Figure 2). Therefore, in the M microbial community system, the main dechlorination products, PCB47, 49, 51, and 53, can be produced by removing chlorine atoms from the meta- and para-positions of PCB101, 110, 138, 149, 153, 158, 174, 180, and 187 (e.g., 234, 245, 2,345, 236, and 2,356, with underlining indicating the chlorine atoms removed during dechlorination) (Supplementary Table S12). On day 112, 62.7% of the meta-chlorine atoms were removed from the M-HM system. In the M-Bla system, only 52.4% of metachlorine atoms were removed (Figure 2). The removal rate of meta-chlorine atoms in the M-HM system was 1.2 times that of the control group (Supplementary Table S12). In the M-HA system, 52.0% of the meta-chlorine atoms were removed, which was very close to the removal rate of the meta-chlorine atoms in M-Bla (52.4%). However, in the M-HA system, 7.0% of the para-chlorine atoms were removed, which was higher than 4.9% in the control group. In the M-AQDS system, on day 112, 4.2% of the para-chlorine atoms were substituted, similar to M-Bla; however, the removal rate of meta-chlorine atoms in M-AQDS was only 50.7% of that in M-Bla.

In the T microbial community system, on Day 0, the total molar percentages of PCB47, 49, 51, and 53 were only 1.7%. After 45 d, the molar percentages of the four PCB congeners in the T-Bla, T-HM, T-HA, and T-AQDS systems were 34.5, 41.5, 19.1, and 8.8%, respectively (Figure 2). In the T microbial community, the main dechlorination products, PCB47, 49, 51, and 53, can be produced by removing metachlorine atoms from PCB135, 138, 149, 153, 174, 180, and 187 (e.g., 234, 235, 236, 245, 2,345, and 2,356). The T microbial community mainly underwent meta-chlorine substitution, with almost no dechlorination occurring at other positions (Supplementary Table S13). After 45 d, the removal rate of meta-chlorine atoms in the T-HM system was 1.1 times that of T-Bla, whereas in the T-HA and T-AQDS systems, the removal rates were only 52.4 and 31.3%, respectively.

### 3.3 Analysis of microbial community structure

**Figure 3:**
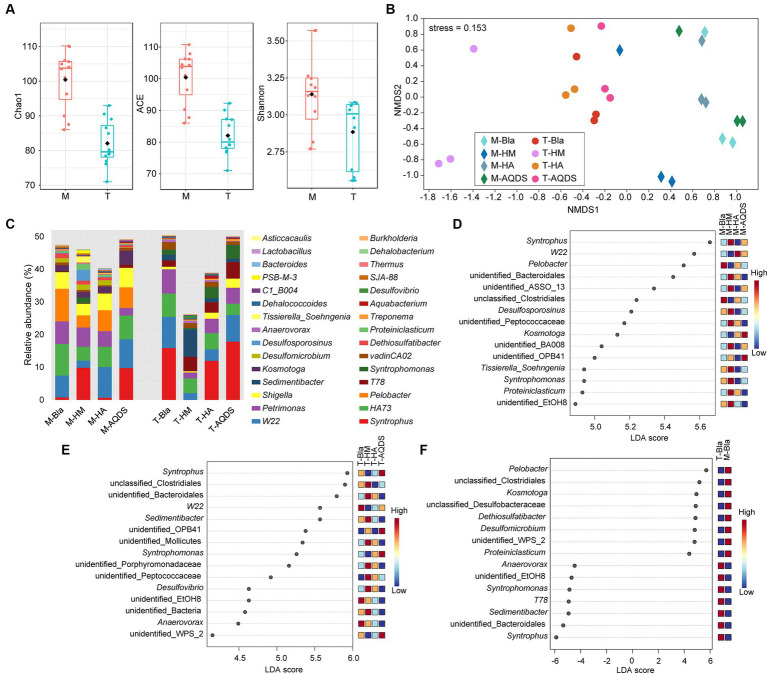
Bacterial community structure and discriminant analysis. **(A)** Alpha diversity indices of the culturing systems enriched from the marine (M) and terrestrial (T) sediments. **(B)** Non-metric multidimensional scaling (NMDS) based on Bray–Curtis of the culturing systems enriched from the marine (M) and terrestrial (T) sediments. **(C)** Bacterial community compositions in the culturing systems enriched from the marine (M) and terrestrial (T) sediments at the genus level. **(D–F)** The analysis of linear discriminant analysis effect size for bacterial community enriched from the marine (M), terrestrial (T) sediment, and between M and T. HM, HA, and AQDS represent the culture systems with humin, humic acid, and anthraquinone-2,6-disulfonic acid, respectively. Bla represents the culture systems without the addition of any electron mediators.

To further understand the impact of different electron mediators on the microbial community structure of dechlorinating bacteria from terrestrial and marine sources of PCBs, high-throughput sequencing was used to characterize the microbial community structure of marine or terrestrial dechlorinating cultures in various treatment systems (Figure 3). Figure 3A shows the alpha diversity of the M-bacterial- and T-bacterial cultures. The Chao1, ACE, and Shannon indices indicated that the richness, evenness, and diversity of the M-bacterial cultures were higher than those of the T-bacterial cultures (*p* < 0.05). Figure 3B shows the NMDS analysis based on the Bray-Curtis distance matrix of the M-bacterial- and T-bacterial cultures. The microbial community structures of marine and terrestrial dechlorinating cultures were significantly different (ANOSIM *R* = 0.552, *p* < 0.05; Adonis *R*^2^ = 0.296, *p* < 0.05). Moreover, the microbial community structures of M-HM (ANOSIM *R* = 0.690, *p* < 0.05; Adonis *R*^2^ = 0.327, *p* < 0.05) and T-HM (ANOSIM *R* = 1, *p* < 0.05; Adonis *R*^2^ = 0.561, *p* < 0.05) differed significantly from those of the other cultivation systems.

Figure 3C presents the microbial community structures of the M-bacterial- and T-bacterial cultures. Firmicutes, Proteobacteria, Bacteroidetes, Synergistetes, and WWE1 were the top five most abundant phyla in both M- and T-dechlorinating cultures, with average relative abundance of 48.41, 18.73, 10.79, 8.29, and 6.31%, respectively. At the genus level (Figure 3C), *Syntrophus* (8.34%), *W22* (6.31%), *HA73* (5.92%), *Petrimonas* (4.8%), and *Pelobacter* (3.3%) were the top five taxa based on average relative abundance. The relative abundances of *Pelobacter*, *Kosmotoga,* and *Dethiosulfatibacter* were significantly higher (Tukey’s HSD; *p* < 0.05) in M-dechlorinating cultures than in T-dechlorinating cultures. Furthermore, the relative abundance of *Dehalococcoides* was significantly higher (Tukey’s HSD; *p* < 0.05) in the AQDS than in the other systems. Specifically, the relative abundance of *Dehalococcoides* in the M-Bla, M-HM, M-HA, and M-AQDS systems was 0.23, 0.19, 0.29, and 0.04%, respectively. The relative abundance of *Dehalococcoides* in the T-Bla, T-HM, T-HA, and T-AQDS systems was 0.21, 0.26, 0.30, and 0.09%, respectively.

LEfSe analysis was conducted at the genus level to investigate the biomarkers of the M- and T-bacterial culture systems, with the top 15 genera showing the highest LDA scores (Figures 3D,E). The differences in community structure between M-HM and other M-bacterial culture systems mainly stem from *syntrophus*, unidentified Bacteroidales, unidentified AssO-13, *Desulfosporosinus*, unidentified Peptococcaceae, unidentified BA008, *Tissierella soehngenia*, *Syntrophomonas*, *Proteiniclasticum*, and unidentified EtOH8. The relative abundances of *Syntrophomonas* and *Desulfosporosinus* in the M-HM system were significantly higher than those in the other M-bacterial culture systems (*p* < 0.05). The differences in community structure between T-HM and other cultivation systems mainly stemmed from unclassified Clostridiales, unidentified Bacteroidales, *Sedimentibacter*, unidentified Mollicutes, unidentified Porphyromonadaceae, unidentified Peptococcaceae, *Desulfovibrio*, and other unidentified bacteria. The relative abundances of *Sedimentibacter* and *Desulfovibrio* in the T-HM system were significantly higher than those in the other T-bacterial culture systems (*p* < 0.05). To explore the differences in community structure between M-bacterial- and T-bacterial cultures, LEfSe analysis at the genus level was conducted for the M-Bla and T-Bla systems (Figure 3F), with the top 15 genera identified at the highest LDA scores. *Pelobacter*, unclassified Clostridiales, *Kosmotoga*, unclassified Desulfobacteraceae, *Dethiosulfatibacter*, *Desulfomicrobium*, unidentified WPS_2, and *Proteiniclasticum* were significantly enriched in the M-bacterial culture systems (*p* < 0.05), whereas *Syntrophus*, unidentified Bacteroidales, *Sedimentibacter*, *T78*, *Syntrophomonas*, unidentified EtOH8, and *Anaerovorax* were significantly enriched in the T-bacterial culture systems (*p* < 0.05).

### 3.4 Co-occurrence network analysis

**Figure 4:**
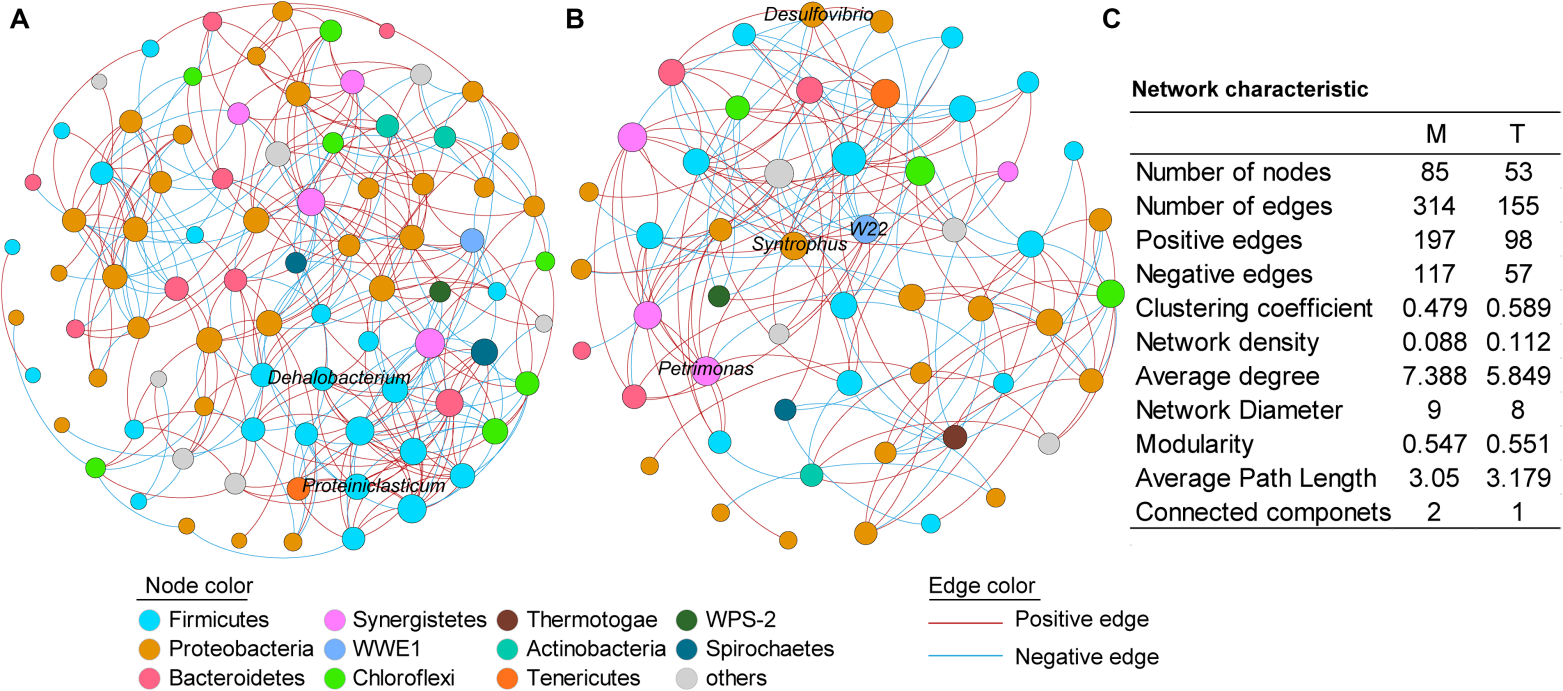
Co-occurrence networks of the culturing systems enriched from the marine **(A)** and terrestrial **(B)** sediments, respectively. **(C)** Topological properties of the co-occurrence networks. M and T represent the PCB dechlorination cultures enriched from terrestrial and marine environments, respectively.

To further investigate the microbial interactions within the anaerobic PCB-dechlorinating bacterial cultures from different terrestrial and marine sources, co-occurrence network analysis was conducted for the M-bacterial- and T-bacterial cultures independently (Figure 4), based on the genera with relative abundance above 0.01%. In the co-occurrence networks, nodes represent bacteria and edges represent statistically significant strong correlations between connected nodes (|r| > 0.7, *p* < 0.05). The number of edges (314 vs. 155) and nodes (85 vs. 53) in the microbial network of the M-dechlorination cultures was much higher than that of the T-dechlorination cultures. Both networks have higher positive correlation ratios. The clustering coefficient, network density, modularity, and average path length in the microbial network of T-dechlorination cultures were higher than those in the M-dechlorination cultures. In contrast, the average degree, network diameter, and connected components in the microbial network of the M dechlorination cultures were higher.

In the network of M-bacterial cultures, the degree of the top five genera with higher relative abundances was between 5–10; while the nodes with higher degrees were unidentified Thermovirgaceae (16), *PSB-M-3* (15), unidentified Eubacteriaceae (15), unidentified Aminiphilaceae (14), and unidentified Bacteroidales (14). In the network of T-bacterial cultures, the degrees of the top five genus were 6–10; while the nodes with higher degrees were *vadinHB04* (14), *C1*_*B004* (10), *Thermus* (10), unidentified Mollicutes (10), and unidentified Aminiphilaceae (10).

### 3.5 Analysis of *Dehalococcoides* and its dechlorination functional genes

**Figure 5:**
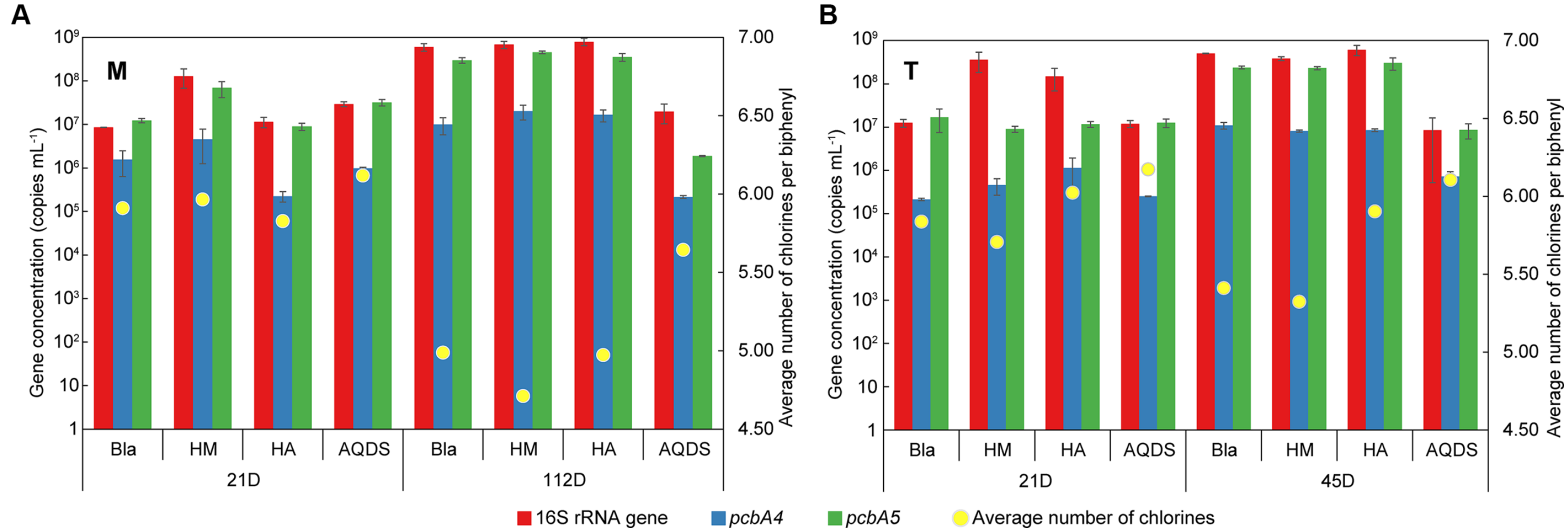
Changes of the gene concentration of *Dehalococcoides* 16S rRNA, *pcbA4,* and *pcbA5* and the average number of chlorines per biphenyl over time in various culturing systems enriched from the marine **(A)** and terrestrial **(B)** sediments. HM, HA, and AQDS represent the culture systems with humin, humic acid, and anthraquinone-2,6-disulfonic acid, respectively. Bla represents the culture systems without the addition of any electron mediators.

To investigate the growth status of *Dehalococcoides*, a potential dechlorinating bacterium mediated by electron shuttles, in different cultivation systems, quantitative PCR (qPCR) was conducted to quantify the 16S rRNA and dechlorination functional genes (*pcbA1*, *pcbA4*, and *pcbA5*) of *Dehalococcoides*. The specific primers CG1-17F and CG1-17R for the dechlorination functional gene *pcbA1* did not amplify in any of the samples, indicating that *pcbA1* was either absent or below the detection limit in the experimental samples. Figure 5 illustrates the temporal changes in the gene concentrations of *Dehalococcoides* 16S rRNA, *pcbA4*, and *pcbA5* in the M-bacterial- and T-bacterial culture cultivation systems.

On day 21, the concentrations of *Dehalococcoides* 16S rRNA gene and dechlorination functional genes in M-Bla, M-HM, M-HA, and M-AQDS were similar (16S rRNA gene concentration ranged from 8.5 × 10^6^ to 1.3 × 10^8^ copies mL^−1^, *pcbA4* gene concentration ranged from 2.3 × 10^5^ to 4.6 × 10^6^, *pcbA5* gene concentration ranged from 9.0 × 10^6^ to 6.9 × 10^7^). Similarly, T-Bla, T-HM, T-HA, and T-AQDS showed the same pattern (16S rRNA gene concentration ranged from 1.2 × 10^7^ to 3.6 × 10^8^ copies mL^−1^, *pcbA4* gene concentration ranged from 2.1 × 10^5^ to 1.1 × 10^6^, *pcbA5* gene concentration ranged from 9.0 × 10^6^ to 1.7 × 10^7^). However, after 112 days of cultivation for M-bacterial cultures and 45 days for T-bacterial cultures, the concentrations of *Dehalococcoides* 16S rRNA gene, *pcbA4,* and *pcbA5* in M-AQDS and T-AQDS were lower than those in other cultivation systems.

**Figure 6:**
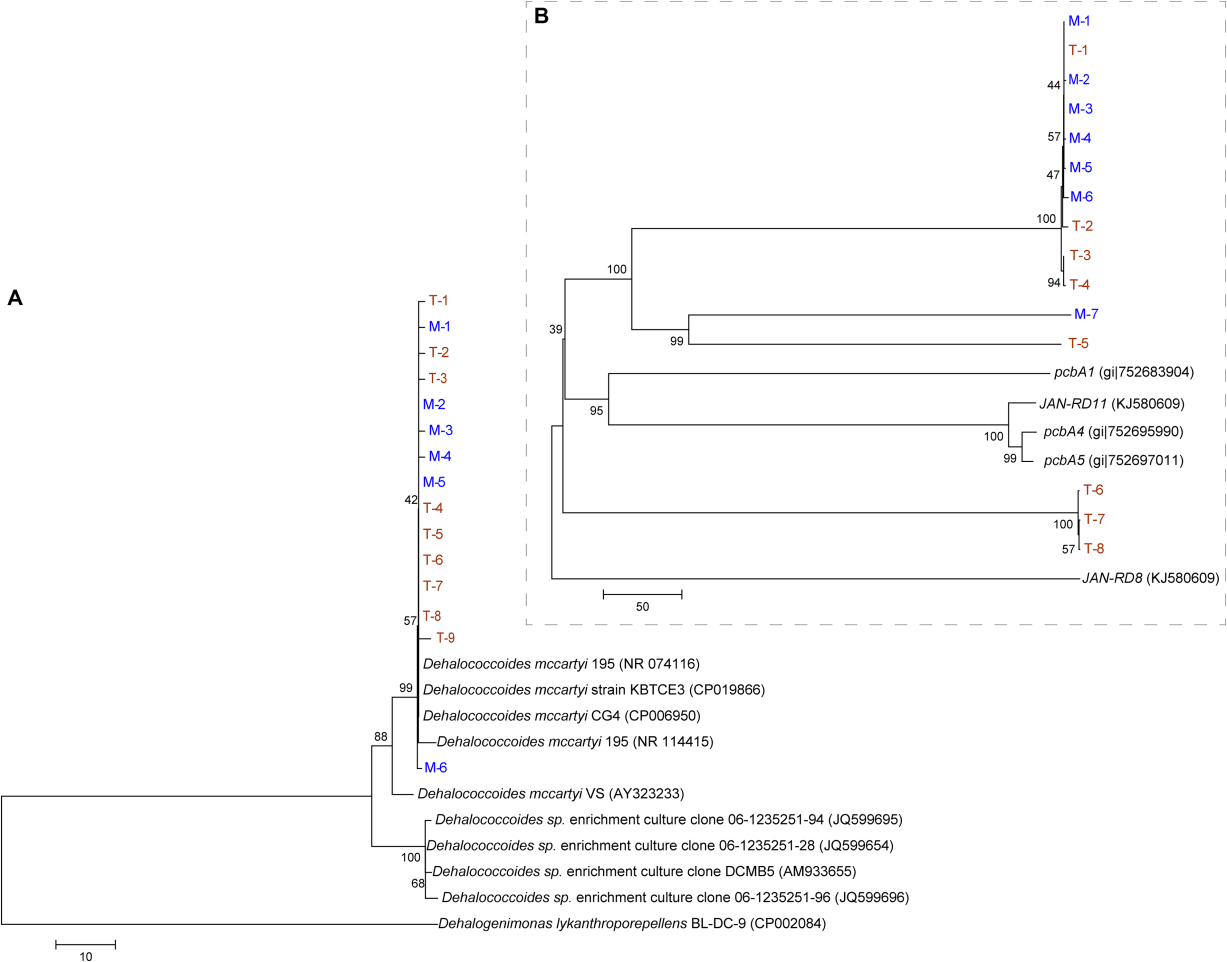
Phylogenetic tree constructed using 16S rRNA gene data **(A)** and reductive dehalogenase gene **(B)** of *Dehalococcoides* from the culture enriched from the marine (M, in blue) and terrestrial (T, in yellow) sediments.

A phylogenetic tree was constructed based on the *Dehalococcoides* 16S rRNA (Figure 6A) and reductive dehalogenase genes (Figure 6B). Figure 6A shows the close phylogenetic relationships among *Dehalococcoides* from different terrestrial and marine sources. Figure 6B shows that the reductive dehalogenase genes in the M and T-bacterial cultures had relatively low sequence similarity to known PCB reductive dehalogenase genes (*pcbA1*, *pcbA4*, *pcbA5*, *JAN-RD8*, and *JAN-RD11*). Moreover, some reductive dehalogenase genes originating from T-bacterial cultures (T-1, T-2, T-3, T-4, and T-5) showed high sequence similarity with those from M-bacterial cultures; however, other reductive dehalogenase genes (T-6, T-7, and T-8) showed relatively low sequence similarity. In this study, T-bacterial cultures exhibited a more diverse array of reductive dehalogenase genes.

## 4 Discussion

### 4.1 Comparative analysis of the effects of humic substances electron mediators on the anaerobic reductive dechlorination activity of PCBs microbial communities from different terrestrial and marine sources

In this study, tetrachlorobiphenyls were the final dechlorination metabolites in all M and T microbial community cultures (Figure 1), which was consistent with other studies (Shah et al., 2013; Wang et al., 2014; Zhang et al., 2017). The lower dechlorination rates observed in the M-Bla, M-HA, and M-AQDS groups on day 15 may be attributed to the fact that these systems were still in a dechlorination lag phase. This is because, at this stage, the number of reductive dehalogenating bacteria in the system is relatively low, and only when the concentration of these bacteria reaches a certain critical level can the entire culture system exhibit dechlorination activity, which often takes a long time (Chen and He, 2018). Moreover, HM promoted the anaerobic reductive dechlorination of PCBs in both M and T microbial communities. M-HM and T-HM showed significant dechlorination activities on day 15 and 21, respectively. The dechlorination rates of M-HM and T-HM were 3.2 times and 1.2 times those of their respective biological control groups. The promoting effect of HM on the M microbial community was stronger than that on the T microbial community. AQDS exhibited inhibitory effects on both the M and T microbial communities. However, the presence of HA had a weak promoting effect on the anaerobic reductive dechlorination of PCBs by the M microbial community but inhibited the activity of the T microbial community.

The redox-active centers of humic substances, such as HM, HA, and AQDS, particularly their internal quinone structures, have been extensively studied (Zhang and Katayama, 2012; Zhang et al., 2017). Quinone structures in humic substances can undergo reduction reactions using electrons from fermenting bacteria (Field and Cervantes, 2005), and these reduced quinone structures can further act as electron donors to promote microbial anaerobic reductive dechlorination metabolism (Field and Cervantes, 2005). Many studies have considered quinone structures crucial for humic substance electron mediators to exert their redox activity and facilitate electron transfer processes (Rau et al., 2002; Doong and Chiang, 2005; Field and Cervantes, 2005).

In this experiment, HM exhibited a stable promoting effect on both M and T microbial communities, whereas HA and AQDS, as humic substances and electron mediators with quinone structures, did not show significant promoting effects. Therefore, solid-state electron mediators showed significant promoting effects on the metabolic activity of PCB dechlorination cultures from different terrestrial and marine sources, whereas dissolved HA and AQDS were not effectively utilized by dechlorination microbial communities for efficient electron transfer, which is consistent with previous research results (Zhang and Katayama, 2012). The solid-state form or specific redox-active structures of HM may play a crucial role in the dechlorination by microbial communities. HM may promote dechlorination in two ways. The HM extracted in this experiment contained various metals, including iron, copper, manganese, zinc, and chromium (Zhang et al., 2017). These metals may form metal–organic complex structures unique to HM, which may mediate electron transfer processes in microbial communities (Zhang and Katayama, 2012). Second, among the three electron mediators, HM is the only solid-state electron mediator that may also affect the anaerobic reductive dechlorination activity of microbial communities. PCBs, as hydrophobic organic pollutants, tend to adsorb onto organic matter such as HM. HM can adsorb organic pollutants through various mechanisms, such as van der Waals forces, hydrogen bonding, and hydrophobic interactions (Lipczynska-Kochany, 2018). Microorganisms can also adhere to HM (Zhang and Katayama, 2012). The adsorption of organic pollutants by HM does not reduce the bioavailability of the adsorbed organic pollutants (Zhang and Katayama, 2012; Zhang et al., 2014). In a static culture medium, the concentration of PCBs in the lower layer of the medium is often higher than that in the upper layer, and HM and dechlorination microbial communities exhibit the same distribution pattern. Therefore, HM may enhance the electron transfer process between microorganisms and HM-PCBs by adsorbing PCBs and providing attachment points for the microorganisms, thereby accelerating the anaerobic reductive dechlorination of PCBs.

HA exhibited a weak promoting effect on the anaerobic reductive dechlorination of PCBs by the M microbial community but significantly inhibited the metabolic activity of the T microbial community. This may be due to differences in the dechlorination characteristics of microbial communities from different environments (the M microbial community is enriched in East China Sea sediments, whereas the T microbial community is enriched in paddy fields), which may produce different responses to the mediation of HA.

### 4.2 Dechlorination pathways of PCBs microbial community from different sources show clear differences

The dominant reductive dechlorination pathways of PCBs microbial communities from different sources were inferred based on profile changes in individual PCB congeners after the cultivation process. First, similar PCB dechlorination metabolites in different experimental groups with the addition of electron mediators indicated that the addition of electron mediators did not affect the extent of reductive dechlorination by microbial communities. Moreover, the M microbial community mainly underwent meta- and para-chlorine dechlorination, whereas the T microbial community exhibited only meta-chlorine dechlorination. Furthermore, both cultures potentially exhibited dechlorination pathways N and H or H′. In the M microbial community, there may exist dechlorination pathways N (e.g., 245, 2,345, and 236) and H or H′ (e.g., 234 and 245), and in the T microbial community, there may exist dechlorination pathways N (e.g., 236, 2,345) and H or H′ (e.g., 234 and 245) (Wiegel and Wu, 2000). Compared to the ortho positions, the chlorine atoms in the meta and para positions were more easily removed, which is consistent with previous reports (Hughes et al., 2010; Zhang et al., 2017).

Furthermore, quantitative changes in chlorines substituted at the ortho-, meta-, and para-positions of the biphenyl rings were compared within various culturing systems after adding humic substances as electron mediators. For the M microbial community (Supplementary Table S12), HA may primarily promote the removal of para-chlorine rather than meta-chlorine because the M microbial community mainly undergoes meta-chlorine dechlorination, which may explain the less pronounced promoting effect of HA. HM primarily promotes the substitution of meta-chlorine to facilitate the anaerobic reductive dechlorination reaction of the M microbial community. AQDS primarily inhibited the removal of meta-chlorine atoms, thereby suppressing the anaerobic reductive dechlorination activity of the M microbial community. In the T microbial community (Supplementary Table S13), HM mainly enhanced the T microbial community’s metabolic response by promoting meta-chlorine dechlorination, whereas HA and AQDS primarily exerted inhibitory effects by suppressing meta-chlorine dechlorination. Therefore, HM primarily enhanced the anaerobic dechlorination metabolic activity of both M and T microbial communities by promoting meta-chlorine dechlorination. HA promotes para-chlorine dechlorination and inhibits meta-chlorine dechlorination, resulting in only a weak promoting effect on the dechlorination metabolism of the M microbial community but an inhibitory effect on the T microbial community, which mainly undergoes meta-chlorine dechlorination. AQDS significantly inhibited meta-chlorine dechlorination, thereby reducing the dechlorination metabolic activity of both the M and T microbial communities. HM, naturally occurring solid-phase organic compounds, have advantages such as minimal environmental impact, low susceptibility to loss, and efficient mediation. Some studies have also reported positive effects of HM on organic pollutants (Zhang and Katayama, 2012; Zhang et al., 2014, 2017). As a solid-phase humic electron mediator, the stable promotion of the anaerobic reductive dechlorination of PCBs expands the application prospects of environmental pollutant bioremediation technology.

### 4.3 HM promotes reductive dechlorination by altering community structure; AQDS inhibits it by restraining dehalococcoides growth

The higher alpha diversity of M-bacterial cultures compared to that of T-bacterial cultures (Figure 3A) may be due to the shorter cultivation time (5 years) and less transfer of M-bacterial cultures compared to T-bacterial cultures (10 years). PCB pollutants in the cultivation system can exert stress on microbial communities, leading to a decrease in the number and diversity of species as cultivation generations progress, resulting in simplification of the microbial ecological structure (Zenteno-Rojas et al., 2020). Longer cultivation times ultimately led to a simpler community structure in T-bacterial cultures than in M-bacterial cultures. NMDS analysis (Figure 3B) indicated that, compared to other humic substances, the addition of HM may significantly alter the community structure of the cultivation system, particularly by potentially altering the relative abundance of key species in the microbial community structure, thereby promoting anaerobic reduction and dechlorination of PCBs.

Currently, *Dehalococcoides*, *Dehalobacterium*, *Dehalogenimonas*, *Dehalobacter*, and *Dehalobium* have been reported to possess PCB dechlorination activity (Li et al., 2014; Zhang et al., 2019). The potential dechlorinating bacteria *Dehalococcoides* and *Dehalobacterium* were detected in all cultivation systems, with average relative abundances of 0.20 and 0.07%, respectively (Figure 3C). *Dehalogenimonas* was detected at a very low relative abundance only in T-AQDS (0.002%), whereas neither *Dehalobacter* nor *Dehalobium* were detected in any of the cultivation systems. Notably, the anaerobic dechlorination activity of *Dehalococcoides* toward PCBs has been widely reported (Wang and He, 2013; Wang et al., 2014, 2019). There was no significant difference in the relative abundance of *Dehalococcoides* between the M-HM and T-HM systems compared with the respective biological control groups. Thus, HM did not promote dechlorination by increasing the relative abundance of *Dehalococcoides*. However, in the M-AQDS and T-AQDS systems, the relative abundance of *Dehalococcoides* was significantly lower than in the corresponding biological control groups (Tukey’s HSD; *p* < 0.05). Therefore, the lower relative abundance of *Dehalococcoides* in the M-AQDS and T-AQDS cultivation systems may have inhibited PCB anaerobic reduction and dechlorination.

LEfSe analysis (Figure 3D) revealed that *Syntrophomonas* and *Desulfosporosinus* were significantly enriched in the M-HM system. Both *Syntrophomonas* and *Desulfosporosinus* have been reported to produce hydrogen (Sieber et al., 2010; Crable et al., 2016; Zhu et al., 2019), which is a necessary electron donor for *Dehalococcoides* involved in PCB anaerobic reduction and dechlorination. Therefore, HM may enhance the metabolic activity of *Dehalococcoides* for PCB dechlorination by increasing the relative abundance of *Syntrophomonas* and *Desulfosporosinus*, thereby strengthening the hydrogen supply. In the T-bacterial cultures, *Sedimentibacter* and *Desulfovibrio* were enriched in the T-HM system (Figure 3E). Certain species of *Sedimentibacter* have been reported to synthesize and provide cobalamins to other genera (Maphosa et al., 2012). Cobalamin is crucial for maintaining the activity of reductive dehalogenases in the OHRB (He et al., 2007). The increase in the relative abundance of *Sedimentibacter* may provide more cobalamin to OHRB, thereby enhancing its PCB anaerobic reduction and dechlorination activity. *Desulfovibrio* can convert lactate to formate and decompose formate into carbon dioxide and hydrogen ions via formate dehydrogenase (Wang et al., 2019). *Desulfovibrio* can convert lactate to acetate. *Dehalococcoides* can utilize acetate as a carbon source, convert it to acetyl-CoA, and enter the tricarboxylic acid cycle. Therefore, *Desulfovibrio* may support the reductive dechlorination by *Dehalococcoides* by providing electron donors (hydrogen) and carbon sources (acetate). The interplay between *Desulfovibrio* and *Dehalococcoides* is crucial for promoting anaerobic PCB reduction and dechlorination. Therefore, the promoting effect of HM on T-bacterial cultures may be achieved by increasing the relative abundances of *Sedimentibacter* and *Desulfovibrio*, thereby enhancing cobalamin, carbon sources, and hydrogen supply. In summary, HM may promote the anaerobic reduction and dechlorination of both M- and T-bacterial cultures by strengthening the hydrogen production capacity in M-bacterial cultures and enhancing the cobalamin, carbon source, and hydrogen supply in T-bacterial cultures. Further research is required to elucidate the specific promoting mechanisms.

LEfSe analysis (Figure 3F) showed that sulfate-reducing bacteria [unclassified-*Desulfobacteraceae* (Kümmel et al., 2015), *Dethiosulfatibacter* (Takii et al., 2007), and *Desulfomicrobium* (Dias et al., 2008)] contributed significantly to the differences in microbial community structure between the M-bacterial- and T-bacterial cultures. This may be because M-bacterial cultures are enriched in sulfate-reducing bacteria owing to the higher sulfate concentration in marine environments where they are predominant. Sulfate-reduction metabolism mediated by sulfate-reducing bacteria in the microbial community may directly affect the metabolic activity of dechlorinating bacteria or influence their metabolic interactions with sulfate-reducing bacteria, thereby affecting the reductive dechlorination metabolism of PCBs by marine-origin dechlorinating bacterial cultures (El Mamouni et al., 2002; Aulenta et al., 2008; Mao et al., 2017). Therefore, the influence of sulfate and its microbial reduction metabolism on the PCB-dechlorination activity of marine-origin dechlorinating bacterial cultures warrants further investigation.

### 4.4 More diverse interspecies interactions existed among marine-derived dechlorinating microbial communities

The co-occurrence network revealed differences in bacterial interspecies interactions among dechlorinating microbial communities from marine and terrestrial sources. Positive correlations (red lines) indicate similar ecological niches or cooperative relationships between connected genera, whereas negative correlations (blue lines) indicate competitive relationships (Lin et al., 2019). Thus, there were more cooperative relationships than competitive relationships among the bacteria in both the M- and T-bacterial culture systems. Moreover, both the M- and T-bacterial culture networks had a modularity greater than 0.5, indicating that both networks exhibited modular structures. In addition, compared to the T-bacterial cultures, the co-occurrence network of the M-bacterial cultures had a greater number of edges and a higher average degree, indicating that the interspecies interactions within the M-bacterial cultures were more diverse than those within the T-bacterial cultures. This may be because the M-bacterial cultures originate from a more complex marine environment. Significant dechlorination activity was observed in both the M-bacterial- and T-bacterial cultures after 15 and 21 days of cultivation, respectively. The greater diversity of interspecies interactions within the M-bacterial cultures may have contributed to their higher anaerobic PCB dechlorination activity.

Notably, the numerical value of the node degree indicates the number of co-occurring interactions between the corresponding genera and other genera (Greenblum et al., 2011). In the co-occurrence network of M-bacterial cultures, *Proteiniclasticum* had a degree of 12, making it one of the highest degree nodes. *Proteiniclasticum*, an obligate anaerobe, can ferment amino acids and proteins to produce acetate (Zhang et al., 2010). *Dehalobacterium*, with a degree of 10, is another node with a relatively high degree and is known to participate in the reductive dechlorination of 4-chlorophenol (Li et al., 2014). In T-bacterial cultures, nodes with high degrees included *W22*, *Syntrophus*, *Petrimonas*, and *Desulfovibrio*. *W22*, with a degree of 9, is one of the highest-degree nodes and has been reported as a potential PCP anaerobic dechlorinating bacterium (Tong et al., 2017). The degrees of *Syntrophus*, *Petrimonas*, and *Desulfovibrio* were 9, 6, and 7, respectively. *Syntrophus* can engage in extracellular electron transfer with other microorganisms (Yan et al., 2018), *Petrimonas* can engage in direct interspecies electron transfer with archaea (Zhao and Zhang, 2019), and *Desulfovibrio* can transfer electrons to other microorganisms through various means, such as cytochromes, pili, and flagella (Zheng et al., 2021). Compared to the M-bacterial cultures, many nodes with higher degrees in the T-bacterial cultures were associated with interspecies electron transfer and occupied important positions. These bacteria may enhance the transfer of electrons between species. However, genera corresponding to nodes with higher degrees in M-bacterial cultures have rarely been reported to participate in interspecies electron transfer. Therefore, compared to the T-bacterial cultures, humic substances, as electron mediators, exhibited a more pronounced promoting effect on the anaerobic PCB dechlorination of the M-bacterial cultures.

### 4.5 Dehalococcoides may be possibly inhibited by AQDS and new PCB-reducing dehalogenase genes may exist

Quantitative PCR showed that the concentrations of the Dehalococcoides 16S rRNA gene and dechlorination functional genes were similar among the different treatments on day 21. Moreover, no significant correlation was found between the concentration of the *Dehalococcoides* 16S rRNA gene and the degree of anaerobic PCB dechlorination in either M-bacterial or T-bacterial cultures (Supplementary Table S14). Therefore, HM does not promote PCB dechlorination by promoting the growth of *Dehalococcoides* as a carbon source. For *pcbA4* and *pcbA5*, there was no significant correlation between their gene concentrations and the degree of anaerobic PCB dechlorination after 21 days of cultivation for both M-bacterial and T-bacterial cultures (Supplementary Table S14). After 112 d of cultivation for M-bacterial cultures and 45 d for T-bacterial cultures, the concentrations of *Dehalococcoides* 16S rRNA gene and dechlorination functional genes in M-AQDS and T-AQDS were lower than those in the control and other treatment groups. Thus, in the late stages of dechlorination, AQDS may inhibit the growth of *Dehalococcoides*, potentially leading to the suppression of PCB dechlorination activity in dechlorinating cultures. Further analysis and comparison of the expression levels of functional genes in each cultivation system are needed for a more in-depth analysis.

Although dechlorinating bacteria *Dehalococcoides* from marine and terrestrial environments are closely related (Figure 6), the low sequence similarity of the reductive dehalogenase genes in this study with known PCB reductive dehalogenase genes indicated that there may be new potential PCB reductive dehalogenase genes in both M and T-bacterial cultures, especially for the marine-derived M-bacterial cultures, as research on PCB reductive dehalogenase genes from marine sources is currently lacking. Therefore, the evolutionary mechanisms of *Dehalococcoides* from different terrestrial and marine sources, and their reductive dehalogenase genes, require further investigation.

## 5 Conclusion

In this study, three types of humic substance electron mediators (HM, HA, and AQDS) were separately added to anaerobic PCB-dechlorinating bacterial cultures from marine (M culture) and terrestrial (T culture) sources. The M culture underwent both meta- and para-chlorine dechlorination, whereas the T culture underwent only meta-chlorine dechlorination. HM primarily enhanced the dechlorination metabolism activity of the cultures by promoting meta-dechlorination, whereas HA exhibited a weak promoting effect on ortho-dechlorination, an inhibitory effect on meta-dechlorination for the M culture, and an inhibitory effect on dechlorination metabolism for the T culture. AQDS exerted inhibitory effects on both cultures by suppressing meta-dechlorination. High-throughput sequencing and qPCR results showed that HM might promote the dechlorination of cultures by altering the community structure. AQDS may hinder the dechlorination of anaerobic PCB-dechlorinating bacterial cultures by inhibiting the growth of *Dehalococcoides*. Co-occurrence network analysis revealed that, compared to the T culture, the M culture exhibited more diverse interspecies interactions, likely contributing to its higher PCB dechlorination activity. Moreover, the taxa involved in interspecies electron transfer played a more significant role in the T culture than in the M culture, possibly explaining why HM enhanced PCB dechlorination more effectively in the M culture than in the T culture. In the future, more in-depth studies can be conducted on the metabolic capabilities and interspecies interactions of dechlorinating bacterial communities, through multi-omics analysis, analysis of the expression levels of dehalogenase genes, and other molecular biology methods.

## Data availability statement

The datasets presented in this study can be found in the NCBI repository, accession number PRJNA1126148.

## Author contributions

QW: Data curation, Formal analysis, Methodology, Software, Visualization, Writing – original draft. DZ: Methodology, Writing – original draft, Conceptualization, Funding acquisition, Investigation, Project administration, Resources, Supervision, Writing – review & editing. XL: Writing – original draft, Writing – review & editing, Data curation, Methodology, Validation, Investigation. YW: Data curation, Formal analysis, Methodology, Writing – review & editing. HW: Data curation, Investigation, Methodology, Resources, Writing – review & editing. ZZ: Formal analysis, Investigation, Methodology, Resources, Software, Writing – review & editing. WS: Conceptualization, Funding acquisition, Project administration, Resources, Supervision, Validation, Writing – review & editing. PG: Conceptualization, Funding acquisition, Project administration, Supervision, Writing – review & editing.

## Footnotes

^1^ https://www.microbiomeanalyst.ca/MicrobiomeAnalyst/

## References

[ref1] Aulenta F. Beccari M. Majone M. Papini M. P. Tandoi V. (2008). Competition for H_2_ between sulfate reduction and dechlorination in butyrate-fed anaerobic cultures. Process Biochem. 43, 161–168. doi: 10.1016/j.procbio.2007.11.006

[ref2] Bastian M. Heymann S. Jacomy M. (2009). “Gephi: an open source software for exploring and manipulating networks”, In: Proceedings of the Third International AAAI Conference on Weblogs and Social Media: AAAI Publications.

[ref3] Bedard D. L. Ritalahti K. M. Löffler F. E. (2007). The *Dehalococcoides* population in sediment-free mixed cultures metabolically dechlorinates the commercial polychlorinated biphenyl mixture Aroclor 1260. Appl. Environ. Microbiol. 73, 2513–2521. doi: 10.1128/AEM.02909-06, PMID: 17308182 PMC1855590

[ref4] Bokulich N. A. Kaehler B. D. Rideout J. R. Dillon M. Bolyen E. Knight R.. (2018). Optimizing taxonomic classification of marker-gene amplicon sequences with QIIME 2’s q2-feature-classifier plugin. Microbiome 6:90. doi: 10.1186/s40168-018-0470-z, PMID: 29773078 PMC5956843

[ref5] Callahan B. J. McMurdie P. J. Rosen M. J. Han A. W. Johnson A. J. A. Holmes S. P. (2016). DADA2: High-resolution sample inference from Illumina amplicon data. Nat. Methods 13, 581–583. doi: 10.1038/nmeth.3869, PMID: 27214047 PMC4927377

[ref6] Caporaso J. G. Lauber C. L. Walters W. A. Berg-Lyons D. Lozupone C. A. Turnbaugh P. J.. (2010). Global patterns of 16S rRNA diversity at a depth of millions of sequences per sample. Proc. Natl. Acad. Sci. USA 108, 4516–4522. doi: 10.1073/pnas.1000080107 20534432 PMC3063599

[ref7] Chen C. He J. (2018). Strategy for the rapid dechlorination of polychlorinated biphenyls (PCBs) by *Dehalococcoides mccartyi s*trains. Environ. Sci. Technol. 52, 13854–13862. doi: 10.1021/acs.est.8b03198, PMID: 30457846

[ref8] Chong J. Liu P. Zhou G. Xia J. (2020). Using MicrobiomeAnalyst for comprehensive statistical, functional, and meta-analysis of microbiome data. Nat. Protoc. 15, 799–821. doi: 10.1038/s41596-019-0264-1, PMID: 31942082

[ref9] Crable B. R. Sieber J. R. Mao X. Alvarez-Cohen L. Gunsalus R. Ogorzalek Loo R. R.. (2016). Membrane complexes of syntrophomonas wolfei involved in syntrophic butyrate degradation and hydrogen formation. Front. Microbiol. 7:1795. doi: 10.3389/fmicb.2016.01795, PMID: 27881975 PMC5101538

[ref10] Dias M. Salvado J. C. Monperrus M. Caumette P. Amouroux D. Duran R.. (2008). Characterization of *Desulfomicrobium salsuginis* sp. nov. and *Desulfomicrobium aestuarii* sp. nov., two new sulfate-reducing bacteria isolated from the Adour estuary (French Atlantic coast) with specific mercury methylation potentials. Syst. Appl. Microbiol. 31, 30–37. doi: 10.1016/j.syapm.2007.09.002, PMID: 18453046

[ref11] Doong R.-A. Chiang H.-C. (2005). Transformation of carbon tetrachloride by thiol reductants in the presence of quinone compounds. Environ. Sci. Technol. 39, 7460–7468. doi: 10.1021/es047956k, PMID: 16245816

[ref12] Eisen J. A. Huse S. M. Dethlefsen L. Huber J. A. Welch D. M. Relman D. A.. (2008). Exploring microbial diversity and taxonomy using SSU rRNA hypervariable tag sequencing. PLoS Genet. 4:e1000255. doi: 10.1371/journal.pgen.1000255, PMID: 19023400 PMC2577301

[ref13] El Mamouni R. Jacquet R. Gerin P. Agathos S. N. (2002). Influence of electron donors and acceptors on the bioremediation of soil contaminated with trichloroethene and nickel: laboratory- and pilot-scale study. Water Sci. Technol. 45, 49–54. doi: 10.2166/wst.2002.0286, PMID: 12188576

[ref14] Fagervold S. K. May H. D. Sowers K. R. (2007). Microbial reductive dechlorination of Aroclor 1260 in baltimore harbor sediment microcosms is catalyzed by three phylotypes within the phylum *Chloroflexi*. Appl. Environ. Microbiol. 73, 3009–3018. doi: 10.1128/AEM.02958-06, PMID: 17351091 PMC1892865

[ref15] Field J. A. Cervantes F. J. (2005). “Microbial redox reactions mediated by humus and structurally related quinones,” in Use of humic substances to remediate polluted environments: From theory to practice. eds. Perminova I. V. Hatfield K. Hertkorn N. (Netherlands: Springer), 343–352.

[ref16] Greenblum S. Turnbaugh P. J. Borenstein E. (2011). Metagenomic systems biology of the human gut microbiome reveals topological shifts associated with obesity and inflammatory bowel disease. Proc. Natl. Acad. Sci. 109, 594–599. doi: 10.1073/pnas.1116053109 22184244 PMC3258644

[ref17] Guo P. Zhang C. Wang Y. Yu X. Zhang Z. Zhang D. (2018). Effect of long-term fertilization on humic redox mediators in multiple microbial redox reactions. Environ. Pollut. 234, 107–114. doi: 10.1016/j.envpol.2017.10.106, PMID: 29172040

[ref18] He J. Holmes V. F. Lee P. K. H. Alvarez-Cohen L. (2007). Influence of Vitamin B12 and cocultures on the growth of *Dehalococcoides* isolates in defined medium. Appl. Environ. Microbiol. 73, 2847–2853. doi: 10.1128/AEM.02574-06, PMID: 17337553 PMC1892872

[ref19] Hughes A. S. VanBriesen J. M. Small M. J. (2010). Identification of structural properties associated with polychlorinated biphenyl dechlorination processes. Environ. Sci. Technol. 44, 2842–2848. doi: 10.1021/es902109w, PMID: 20025283

[ref20] Ihaka R. Gentleman R. (1996). R: A language for data analysis and graphics. J. Comput. Graph. Stat. 5, 299–314. doi: 10.1080/10618600.1996.10474713

[ref21] Kumar S. Stecher G. Tamura K. (2016). MEGA7: Molecular Evolutionary Genetics Analysis Version 7.0 for Bigger Datasets. Mol. Biol. Evol. 33, 1870–1874. doi: 10.1093/molbev/msw054, PMID: 27004904 PMC8210823

[ref22] Kümmel S. Herbst F.-A. Bahr A. Duarte M. Pieper D. H. Jehmlich N.. (2015). Anaerobic naphthalene degradation by sulfate-reducing Desulfobacteraceae from various anoxic aquifers. FEMS Microbiol. Ecol. 91:fiv006. doi: 10.1093/femsec/fiv006 25764566

[ref23] Li Z. Suzuki D. Zhang C. Yang S. Nan J. Yoshida N.. (2014). Anaerobic 4-chlorophenol mineralization in an enriched culture under iron-reducing conditions. J. Biosci. Bioeng. 118, 529–532. doi: 10.1016/j.jbiosc.2014.04.007, PMID: 24794625

[ref24] Lin X.-Q. Li Z.-L. Liang B. Zhai H.-L. Cai W.-W. Nan J.. (2019). Accelerated microbial reductive dechlorination of 2,4,6-trichlorophenol by weak electrical stimulation. Water Res. 162, 236–245. doi: 10.1016/j.watres.2019.06.068, PMID: 31279315

[ref25] Lipczynska-Kochany E. (2018). Humic substances, their microbial interactions and effects on biological transformations of organic pollutants in water and soil: A review. Chemosphere 202, 420–437. doi: 10.1016/j.chemosphere.2018.03.104, PMID: 29579677

[ref26] Mao X. Polasko A. Alvarez-Cohen L. Kelly R. M. (2017). Effects of sulfate reduction on trichloroethene dechlorination by *Dehalococcoides*-containing microbial communities. Appl. Environ. Microbiol. 83:e03384-16. doi: 10.1128/AEM.03384-16, PMID: 28159790 PMC5377507

[ref27] Maphosa F. van Passel M. W. J. de Vos W. M. Smidt H. (2012). Metagenome analysis reveals yet unexplored reductive dechlorinating potential of *Dehalobacter* sp. E1 growing in co-culture with *Sedimentibacter* sp. Environ. Microbiol. Rep. 4, 604–616. doi: 10.1111/j.1758-2229.2012.00376.x, PMID: 23760931

[ref28] Pham D. M. Kasai T. Yamaura M. Katayama A. (2021). Humin: No longer inactive natural organic matter. Chemosphere 269:128697. doi: 10.1016/j.chemosphere.2020.128697, PMID: 33139048

[ref29] Rau J. Knackmuss H.-J. Stolz A. (2002). Effects of different quinoid redox mediators on the anaerobic reduction of azo dyes by bacteria. Environ. Sci. Technol. 36, 1497–1504. doi: 10.1021/es010227+, PMID: 11999057

[ref30] Shah V. Wang S. He J. (2013). Phylogenetically distinct bacteria involve extensive dechlorination of Aroclor 1260 in sediment-free cultures. PLoS One 8:e59178. doi: 10.1371/journal.pone.0059178, PMID: 23554991 PMC3598663

[ref31] Sieber J. R. Sims D. R. Han C. Kim E. Lykidis A. Lapidus A. L.. (2010). The genome of *Syntrophomonas wolfei*: new insights into syntrophic metabolism and biohydrogen production. Environ. Microbiol. 12, 2289–2301. doi: 10.1111/j.1462-2920.2010.02237.x, PMID: 21966920

[ref32] Takii S. Hanada S. Tamaki H. Ueno Y. Sekiguchi Y. Ibe A.. (2007). *Dethiosulfatibacter aminovorans* gen. nov., sp. nov., a novel thiosulfate-reducing bacterium isolated from coastal marine sediment via sulfate-reducing enrichment with Casamino acids. Int. J. Syst. Evol. Microbiol. 57, 2320–2326. doi: 10.1099/ijs.0.64882-0 17911304

[ref33] Tong H. Chen M. Li F. Liu C. Liao C. (2017). Changes in the microbial community during repeated anaerobic microbial dechlorination of pentachlorophenol. Biodegradation 28, 219–230. doi: 10.1007/s10532-017-9791-z, PMID: 28357551

[ref34] Van der Zee F. P. Cervantes F. J. (2009). Impact and application of electron shuttles on the redox (bio)transformation of contaminants: A review. Biotechnol. Adv. 27, 256–277. doi: 10.1016/j.biotechadv.2009.01.004, PMID: 19500549

[ref35] Wang S. Chen C. Zhao S. He J. (2019). Microbial synergistic interactions for reductive dechlorination of polychlorinated biphenyls. Sci. Total Environ. 666, 368–376. doi: 10.1016/j.scitotenv.2019.02.283 30798243

[ref36] Wang S. Chng K. R. Wilm A. Zhao S. Yang K.-L. Nagarajan N.. (2014). Genomic characterization of three unique *Dehalococcoides* that respire on persistent polychlorinated biphenyls. Proc. Natl. Acad. Sci. 111, 12103–12108. doi: 10.1073/pnas.1404845111, PMID: 25028492 PMC4142991

[ref37] Wang S. He J. (2013). Dechlorination of commercial pcbs and other multiple halogenated compounds by a sediment-free culture containing *Dehalococcoides* and *Dehalobacter*. Environ. Sci. Technol. 47, 130904143020001–130904143010534. doi: 10.1021/es4017624, PMID: 23964900

[ref38] Wiegel J. Wu Q. (2000). Microbial reductive dehalogenation of polychlorinated biphenyls. FEMS Microbiol. Ecol. 32, 1–15. doi: 10.1111/j.1574-6941.2000.tb00693.x, PMID: 10779614

[ref39] Yan W. Sun F. Liu J. Zhou Y. (2018). Enhanced anaerobic phenol degradation by conductive materials via EPS and microbial community alteration. Chem. Eng. J. 352, 1–9. doi: 10.1016/j.cej.2018.06.187

[ref40] Zanaroli G. Negroni A. Häggblom M. M. Fava F. (2015). Microbial dehalogenation of organohalides in marine and estuarine environments. Curr. Opin. Biotechnol. 33, 287–295. doi: 10.1016/j.copbio.2015.03.013, PMID: 25863015

[ref41] Zenteno-Rojas A. Martínez-Romero E. Castañeda-Valbuena D. Rincón-Molina C. I. Ruíz-Valdiviezo V. M. Meza-Gordillo R.. (2020). Structure and diversity of native bacterial communities in soils contaminated with polychlorinated biphenyls. AMB Express 10:124. doi: 10.1186/s13568-020-01058-8, PMID: 32651884 PMC7351888

[ref42] Zhang D. Dang H. Li Z. Zhang C. (2019). Redox characteristics of humins and their coupling with potential PCB dechlorinators in southern Yellow Sea sediments. Environ. Pollut. 252, 296–304. doi: 10.1016/j.envpol.2019.05.121, PMID: 31158658

[ref43] Zhang C. Katayama A. (2012). Humin as an electron mediator for microbial reductive dehalogenation. Environ. Sci. Technol. 46, 6575–6583. doi: 10.1021/es3002025, PMID: 22582856

[ref44] Zhang K. Song L. Dong X. (2010). *Proteiniclasticum ruminis* gen. nov., sp. nov., a strictly anaerobic proteolytic bacterium isolated from yak rumen. Int. J. Syst. Evol. Microbiol. 60, 2221–2225. doi: 10.1099/ijs.0.011759-0 19915115

[ref45] Zhang D. Zhang C. Li Z. Suzuki D. Komatsu D. D. Tsunogai U.. (2014). Electrochemical stimulation of microbial reductive dechlorination of pentachlorophenol using solid-state redox mediator (humin) immobilization. Bioresour. Technol. 164, 232–240. doi: 10.1016/j.biortech.2014.04.071, PMID: 24859215

[ref46] Zhang D. Zhang N. Yu X. Zhang Z. Yang S. Zhang C. (2017). Effect of humins from different sediments on microbial degradation of 2,2′,4,4′,5,5′-hexachlorobiphenyl (PCB153), and their polyphasic characterization. RSC Adv. 7, 6849–6855. doi: 10.1039/C6RA25934K

[ref47] Zhao Z. Zhang Y. (2019). Application of ethanol-type fermentation in establishment of direct interspecies electron transfer: A practical engineering case study. Renew. Energy 136, 846–855. doi: 10.1016/j.renene.2019.01.055

[ref48] Zheng S. Li M. Liu Y. Liu F. (2021). *Desulfovibrio* feeding *Methanobacterium* with electrons in conductive methanogenic aggregates from coastal zones. Water Res. 202:117490. doi: 10.1016/j.watres.2021.117490, PMID: 34364064

[ref49] Zhu M. Zhang L. Franks A. E. Feng X. Brookes P. C. Xu J.. (2019). Improved synergistic dechlorination of PCP in flooded soil microcosms with supplementary electron donors, as revealed by strengthened connections of functional microbial interactome. Soil Biol. Biochem. 136:107515. doi: 10.1016/j.soilbio.2019.06.011

