# Supplementary material for: Effects of humic electron mediators on reductive dechlorination of polychlorinated biphenyl by consortia enriched from terrestrial and marine environments
Source: Front Microbiol. 2024 Aug 1;15:1452787. doi: 10.3389/fmicb.2024.1452787 (PMC11324565; doi:10.3389/fmicb.2024.1452787)
Supplement: Supplementary file 1 [file Table_1.DOCX]

**Supplementary Tables**

**Table S1.** The physicochemical properties and metal concentrations of humin (HM).

|  | Element | Content |
| --- | --- | --- |
| Elemental Composition (%) | C  H  N  S  Fe  Cu  Mn | 1.51  1.19  0.09  0.12  10.56  0.52  0.34 |
| Content of metal (mg g^-1^ humin) | Zn  Cr  Cd  Pb | 0.10  0.07  N.D.  N.D. |

N.D. indicates that the value is below the detection line.

**Table S2.** 100-fold concentrated salts stock solution.

| Composition of the Salts stock solution (100 ×) | Add amount of 1 L (g) |
| --- | --- |
| NaCl | 100.00 |
| MgCl_2_·6H_2_O | 50.00 |
| KH_2_PO_4_ | 20.00 |
| NH_4_Cl | 74.00 |
| KCl | 30.00 |
| CaCl_2_·2H_2_O | 1.20 |

**Table S3.** 10000-fold concentrated Se/W stock solution.

| Composition of the Se/W stock solution (10000 ×) | Add amount of 1 L (g) |
| --- | --- |
| NaOH | 5.00 |
| Na_2_WO_4_·2H_2_O | 0.08 |
| NaSeO_3_·5H_2_O | 0.06 |

**Table S4.** 2000-fold concentrated Trace element stock solution.

| Composition of trace element stock solution (2000 ×) | Add amount of 1 L (g) | Add amount of 1 L (mL) |
| --- | --- | --- |
| FeCl_2_·4H_2_O  CoCl_2_·6H_2_O  MnCl_2_·4H_2_O  ZnCl_2_  Na_2_MoO_4_·2H_2_O  NiCl_2_·6H_2_O  H_3_BO_3_  CuCl_2_·2H_2_O  HCl (25%, ω) | 3.00  0.38  0.20  0.14  0.072  0.040  0.012  0.004 | 20.00 |

**Table S5.** 100-fold concentrated reducing agents stock solution.

| Composition of reducing agent stock solution (100 ×) | Add amount of 1 L (g) |
| --- | --- |
| L-cysteinate | 3.14 |
| Na_2_S·9H_2_O | 4.80 |
| Thiosemicarbazone | 7.71 |

**Table S6.** 1000-fold concentrated Vitamin stock solution.

| Composition of vitamin stock solution (1000 ×) | Add amount of 1 L (g) |
| --- | --- |
| Thiamine | 0.20 |
| Niacin | 0.20 |
| Para-aminobenzoic acid | 0.20 |
| Lipoic acid | 0.20 |
| Scaldol hydrochloride | 0.40 |
| Vitamin B12 | 0.004 |
| +D-pantothenic acid | 0.20 |
| +Niacinamide | 0.20 |
| Biotin | 0.08 |

**Table S7.** The component of anaerobic medium before sterilization.

| Composition of anaerobic medium before autoclaving | Add amount of 1 L |
| --- | --- |
| Salts stock solution (100 ×) | 10 mL |
| Se/W stock solution (10000 ×) | 0.1 mL |
| Trace element stock solution (2000 ×) | 0.5 mL |
| 3-morphopropionic acid (Buffers) | 10 mmol |
| Blade Skygreen (0.1% solution, Anaerobic Indicator) | 1 mL |
| NaHCO^­^_3_ | 30 mmol |
| Sodium lactate (Carbon Source) | 10 mmol |
| ddH_2_O | Fill up to 1 L |

**Table S8.** The temperature program of the Gas chromatography-mass spectrometry (GC-MS) column.

| Heating rate (℃/min) | End temperature (℃) | Time (min) |
| --- | --- | --- |
| - | 80.0 | 2.0 |
| 8.0 | 196.0 | 0.0 |
| 2.0 | 228.0 | 0.0 |
| 8.0 | 250.0 | 12.0 |
| 10.0 | 296.0 | 3.0 |
| 10.0 | 300.0 | 2.0 |

**Table S9.** The sequences of primers used in this study.

| Target genes | Primers | Sequence (5′-3′) | References |
| --- | --- | --- | --- |
| *Dehalococcoides*  16S rRNA gene | Dhc-1f | GATGAACGCTAGCGGCG | Yan et al., 2009 |
|  | Dhc-259r | CAGACCAGCTACCGATCGAA |  |
| *Dehalococcoides*  16S rRNA Full length of the gene | Dhc-1f | GATGAACGCTAGCGGCG | Yan et al., 2009; Mészáros et al., 2013 |
|  | Dhc-1377 | GGTTGGCACATCGACTTCAA |  |
| *Dehalogenimonas*  16S rRNA gene | BL-DC-631F | GGTCATCTGATACTGTTGGACTTGAGTATG | Yan et al., 2009 |
|  | BL-DC-796R | ACCCAGTGTTTAGGGCGTGGACTACCAGG |  |
| *Dehalobacter*  16S rRNA gene | Dhb477F | GATTGACGGTACCTAACGAGG | Grostern and Edwards, 2006 |
|  | Dhb647R | TACAGTTTCCAATGCTTTACGG |  |
| *Dehalobium*  16S rRNA gene | 14F | AGAGTTTGATCCTGGCTCAG | Watts et al., 2005 |
|  | Dehal1265R | GCTATTCCTACCTGCTGTACC |  |
| *pcbA1* | CG1-17F | CCGTCAATGGCACTCTGTTCCTTC | Wang et al., 2014 |
|  | CG1-17R | TGCTGGCTTCATTCTCGAAGATCAG |  |
| *pcbA4* | CG4-1F | GGCACAGATGCCTCAAGGAACATAC | Wang et al., 2014 |
|  | CG4-1R | TTGTCCGGCTGCTCCGTCAG |  |
| *pcbA5* | CG5-1F | TGACCAAGGATCTGGTGGAAGGTTG | Wang et al., 2014 |
|  | CG5-1R | AGAAGCGCAATGCCTGAGTGATC |  |
| *Dehalococcoides*  The full length of  reductive dehalogenase gene | RR2F | SHMGBMGWGATTTYATGAARR | Krajmalnik-Brown et al., 2004 |
|  | B1R | CHADHAGCCAYTCRTACCA |  |

**Table S10.** The reaction system of PCR in this study.

| Composition | Volume (μL) | Ultimate concentration (μM) |
| --- | --- | --- |
| DNA template | 1 | - |
| Upstream primer | 1 | 0.2 |
| Downstream primer | 1 | 0.2 |
| Mix | 25 | 1 × |
| ddH_2_O | 22 | - |
| Total | 50 | - |

**Table S11**. The reaction system of qPCR in this study.

| Composition | Volume (μL) | Ultimate concentration (μM) |
| --- | --- | --- |
| DNA template | 1 | - |
| Upstream primer | 0.4 | 0.2 |
| Downstream primer | 0.4 | 0.2 |
| Mix | 10 | 1 × |
| ddH_2_O | 8.2 | - |
| Total | 20 | - |

**Table S12.** Quantitative changes in chlorines substituted in ortho-, meta-, and para-positions of biphenyl rings in various culturing systems enriched from the marine sediment (M).

| **Position of the chlorine atom** | Day 0 | M-Bla-  15D | M-HM-  15D | M-HA-  15D | M-  AQDS-  15D | M-Bla-  21D | M-HM-  21D | M-HA-  21D | M-Bla -  AQDS-  21D | M-Bla-  112D | M-HM-  112D | M-HA-  112D | M-  AQDS-  112D |
| --- | --- | --- | --- | --- | --- | --- | --- | --- | --- | --- | --- | --- | --- |
| Adjacent position | 2.5 | 2.5 | 2.4 | 2.4 | 2.4 | 2.5 | 2.5 | 2.5 | 2.4 | 2.4 | 2.5 | 2.4 | 2.4 |
| Intermediate position | 2.5 | 2.5 | 2.2 | 2.4 | 2.5 | 2.0 | 2.1 | 2.0 | 2.4 | 1.2 | 0.9 | 1.2 | 1.9 |
| Alignment | 1.4 | 1.4 | 1.4 | 1.4 | 1.4 | 1.4 | 1.4 | 1.4 | 1.3 | 1.4 | 1.3 | 1.3 | 1.4 |
| Total | 6.4 | 6.3 | 6.0 | 6.2 | 6.3 | 5.9 | 6.0 | 5.8 | 6.1 | 5.0 | 4.7 | 5.0 | 5.6 |

| **Volume of change (%)** | Day 0 | M-Bla-  15D | M-HM-  15D | M-HA-  15D | M-  AQDS-  15D | M-Bla-  21D | M-HM-  21D | M-HA-  21D | M-Bla -  AQDS-  21D | M-Bla-  112D | M-HM-  112D | M-HA-  112D | M-  AQDS-  112D |
| --- | --- | --- | --- | --- | --- | --- | --- | --- | --- | --- | --- | --- | --- |
| Adjacent position | - | - | - | - | - | - | - | - | - | - | - | - | - |
| Intermediate position | - | -2.8 | - 14.7 | -5.6 | - | - 19.8 | - 17.5 | -21.4 | -6.0 | -52.4 | -62.7 | -52.0 | -26.0 |
| Alignment | - | - | - | - | - | - | - | -3.5 | -7.7 | -4.9 | -8.4 | -7.0 | -4.2 |
| Total | - | -2. 1 | -6.8 | -3.5 | -2. 1 | -8.0 | -7. 1 | -9.3 | -4.8 | -22.4 | -26.7 | -22.6 | - 12. 1 |

**Table S13.** Quantitative changes in chlorines substituted in ortho-, meta-, and para positions of biphenyl rings in various culturing systems enriched from the terrestrial sediment (T).

| **Position of the chlorine atom** | Day 0 | T-Bla-21D | T-HM-21D | T-HA-21D | T-AQDS-21D | T-Bla-45D | T-HM-45D | T-HA-45D | T-AQDS-45D |
| --- | --- | --- | --- | --- | --- | --- | --- | --- | --- |
| Adjacent position | 2.5 | 2.5 | 2.5 | 2.5 | 2.5 | 2.6 | 2.6 | 2.5 | 2.5 |
| Intermediate position | 2.6 | 2.0 | 1.8 | 2.2 | 2.4 | 1.5 | 1.4 | 2.0 | 2.2 |
| Alignment | 1.4 | 1.4 | 1.4 | 1.4 | 1.3 | 1.3 | 1.3 | 1.4 | 1.4 |
| Total | 6.4 | 5.8 | 5.7 | 6.0 | 6.2 | 5.4 | 5.3 | 5.9 | 6.1 |

| **Volume of change (%)** | Day 0 | T-Bla-21D | T-HM-21D | T-HA-21D | T-AQDS-21D | T-Bla-45D | T-HM-45D | T-HA-45D | T-AQDS-45D |
| --- | --- | --- | --- | --- | --- | --- | --- | --- | --- |
| Adjacent position | - | - | - | - | - | - | - | - | - |
| Intermediate position | - | -23. 1 | -29.8 | -21. 1 | -5.9 | -40.4 | -43.5 | -21.2 | - 12.2 |
| Alignment | - | - | - | - | -6.4 | - | - | - | - |
| Total | - | -9.2 | - 11.2 | -6.3 | -4.0 | - 15.8 | - 17.2 | -8.2 | -5.0 |

**Table S14.** The Pearson correlations (r) and their significance (P) between the gene concentrations and the average number of chlorines per biphenyl of the culturing systems enriched from the marine (M) and terrestrial (T) sediments on day 21.

|  | Target gene | Y culturing systems | S culturing systems |
| --- | --- | --- | --- |
| Pearson correlation coefficient (r) | 16S rRNA gene | 0.2 | -0.7 |
|  | *pcbA4* | 0.2 | 0.1 |
|  | *pcbA5* | 0.4 | 0.2 |
| Significance (*P*) | 16S rRNA gene | 0.8 | 0.3 |
|  | *pcbA4* | 0.9 | 0.9 |
|  | *pcbA5* | 0.6 | 0.8 |

**Supplementary Figures**

**Figure S1.** The locations of the sediments used for enriching cultures.


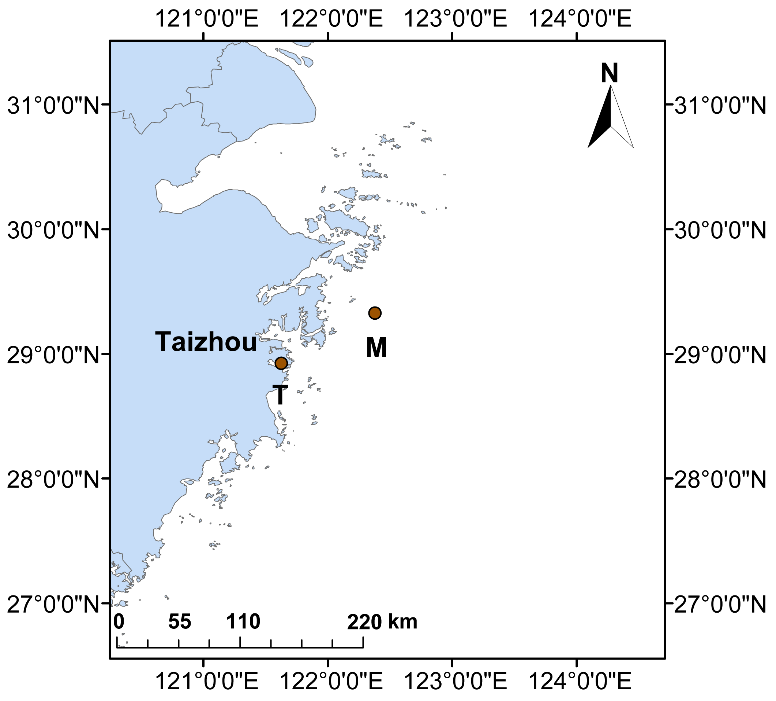


**Figure S2**. The mole percentages of PCBs with different chlorination degrees and the average number of chlorines per biphenyl of the abiotic controls for various culture systems enriched from the marine **(A)** and terrestrial **(B)** sediment, respectively. Abio-Bla represents the abiotic control without the addition of any humic substance electron mediators, Abio-HM represents the abiotic control with the addition of HM, Abio-HA represents the abiotic control with the addition of HA, and Abio-AQDS represents the abiotic control with the addition of AQDS.


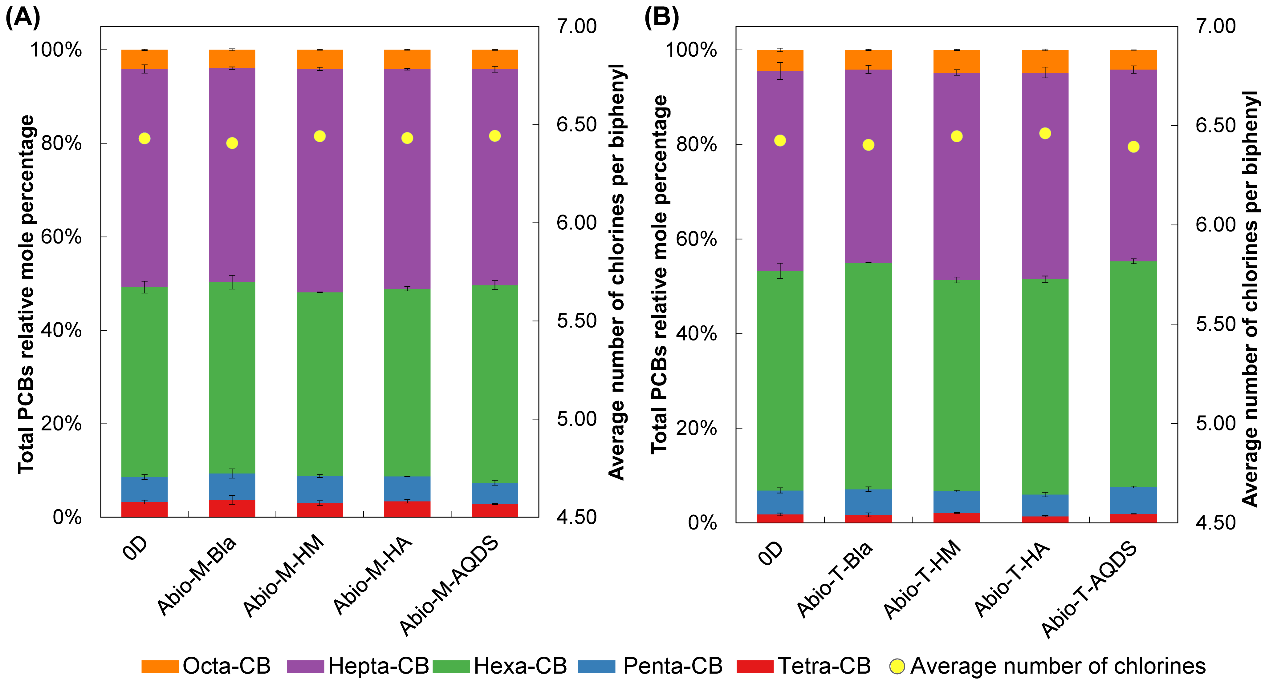


**Supplementary** **Materials and Methods**

**Anaerobic culture medium preparation**

The specific preparation method for anaerobic culture medium is as follows:

1) Prepare Salts Stock Solution, Se/W Stock Solution, Trace Elements Stock Solution, Reducing Agent Stock Solution, and Vitamin Stock Solution according to the formulas in Supplementary Tables S2, S3, S4, S5, and S6, respectively. After preparing the Vitamin Stock Solution, remove oxygen from the solution by purging with nitrogen gas and store it sealed in serum bottles. When preparing the Reducing Agent Stock Solution, boil distilled water to remove oxygen, then quickly add the measured L-cysteine hydrochloride, Na_2_S.9H_2_O, and dithiothreitol, and seal tightly with a rubber stopper. Store all stock solutions at 4°C after preparation.

2) Mix the components listed in Supplementary Table S7 in distilled water and bring the volume up to 1 L. Dispense into serum bottles, with each bottle containing 30 mL of medium. For HM and HA, which can undergo high-temperature sterilization, add them to the anaerobic culture medium at this step. For the experimental group with HM added, add 0.2 g of HM to each serum bottle (final concentration approximately 6.67 g/L). For the experimental group with HA added, add 0.5 mL of HA stock solution to each serum bottle (final concentration of HA is 0.125 g/L).

3) Purge with high-purity nitrogen gas. When the medium changes from deep blue to purple, quickly seal with a butyl rubber stopper coated with polytetrafluoroethylene and seal with aluminum foil.

4) Place the serum bottles containing the anaerobic culture medium into a high-pressure sterilizer for high-pressure sterilization. After the sterilizer cools down, remove the serum bottles.

5) Place the sterilized medium in an anaerobic workstation (AW300SG, Electrotek, Leeds, UK). Add 0.3 mL of the reducing agent stock solution to each serum bottle and shake well. After incubating for 24 hours, if the medium becomes colorless, it is used for the next step of the experiment. The reducing agent stock solution is filtered through a 0.22 μm sterile filter membrane (Merck KGaA, Darmstadt, Germany) before adding it to the medium.

6) Add the corresponding volume of vitamin stock solution (1000 ×) to the colorless medium. The vitamin stock solution is filtered through a 0.22 μm sterile filter membrane before adding it to the medium. For the experimental group with AQDS added, add 1.25 mL of AQDS stock solution (final concentration of AQDS is 1 mM) through a 0.22 μm sterile filter membrane at this step.

**Extraction and analysis for PCBs**

The extraction method for PCBs was as follows:

1) Shake the culture in the serum bottle in the anaerobic workstation, and use a disposable syringe to take out 1 mL of the culture and place it in a 10 mL ground glass tube.

2) Add an equal volume of chromatographically pure n-hexane to the glass tube, vortex for 3 minutes, and then place the glass tube in an ultrasonic cleaner for 20 min.

3) Transfer the upper organic phase to a new 10 mL ground glass tube and add an equal volume of concentrated sulfuric acid, vortex to remove organic phase impurities. Repeat steps 2 and 3 twice.

4) Transfer the collected organic phase to a new glass tube and purge with high-purity nitrogen gas at 30°C. After the n-hexane solution is completely dried, add another 1 mL of chromatographic-grade n-hexane to extract the PCBs and transfer it to an amber liquid-phase vial.

PCBs were qualitatively and quantitatively analyzed using GC-MS (QP2020, Shimadzu, Kyoto, Japan) equipped with an SH-Rxi-5Sil MS column (Shimadzu, Kyoto, Japan). High-purity helium gas was used as the carrier gas. The GC-MS injection port temperature was maintained at 280°C, with a column pressure of 65 kPa, a column flow rate of 1 mL/min, and a linear velocity of 33.6 cm/s. During quantitative analysis, GC-MS operated in Selected Ion Monitoring (SIM) mode. The temperature program is shown in Supplementary Table S8.

By comparing the retention time and integrated ion signals of Aroclor 1260 and its metabolites with a mixture of nine PCBs congeners standard (Accustandard, New Haven, CT, USA, containing 209 congeners), the qualitative analysis of each PCB congener in the samples was completed. Standard curves were constructed by diluting the mixed standard solution gradiently for quantification of Aroclor 1260 and its metabolites using an external standard method. It is generally accepted that under anaerobic conditions, dechlorinating microbial cultures primarily remove chlorine atoms from PCBs without significant degradation of the benzene ring (Shah et al., 2013). Therefore, during the dechlorination process, only the transformation between different chlorinated PCB congeners occurs, while the total mass of PCBs remains constant (Shah et al., 2013). The anaerobic dechlorination activity of PCBs in the culture system was quantitatively analyzed by calculating the molar percentage of each PCB congener and the average number of chlorine atoms per PCB molecule. Only detected PCB congeners were presented and analyzed. The molar percentage of PCB congeners with different chlorine contents was used to calculate the average number of chlorine atoms per PCB molecule in the culture system. The PCB dechlorination rates in the culture system were calculated based on the average number of chlorine atoms per PCB molecule.

**Real-Time Quantitative PCR and Phylogenetic Tree Construction**

PCR reactions were conducted using the 2×EasyTaq®PCR superMix kit (TransGen, Beijing) according to the manufacturer’s instructions. The primer sequences are shown in Supplementary Table S9, and the PCR reaction system is shown in Supplementary Table S10.

The PCR amplification program follows the following temperature and time settings:

1) Initial denaturation: 94°C, maintained for 5 min.

2) Denaturation at 94°C (30 s), annealing (55-60°C, 30 s, optimal annealing temperature determined by primers), extension (72°C, 1 min). This step is repeated for a total of 30 cycles.

3) Final extension at 72°C, maintained for 10 min.

Following PCR amplification, agarose gel electrophoresis (1% concentration) is performed to separate the PCR products, and the lengths of the PCR products are confirmed using a gel imaging system. The desired PCR products meeting the requirements are then excised and recovered using the Easypure® Quick Gel Extraction kit (TransGen Biotech, Beijing) according to the manufacturer's instructions. Subsequently, the recovered target gene PCR products are ligated into vectors using the PEAsY^®^-T1 cloning kit (TransGen Biotech, Beijing) according to the manufacturer’s instructions, and the constructed vectors are transformed into competent cells (Escherichia coli). Positive E. coli colonies are selected through blue-white screening and colony PCR, followed by culture. Fresh bacterial cultures are sent to Shanghai Sangon Biotech Co., Ltd. for Sanger sequencing to confirm the DNA sequences inserted into the vectors as the target gene sequences. The qualified E. coli strains are inoculated into fresh culture medium for cultivation, and plasmids are extracted using the Easypure® plasmid Miniprep kit (TransGen Biotech, Beijing) according to the manufacturer’s instructions to obtain plasmids carrying the target genes. The concentration (ng/μL) and purity of the extracted plasmid DNA are measured using a spectrophotometer (BioTek Instruments, Inc., USA, model: synergyH1). The DNA concentration is converted to copies/μL using the conversion formula: DNA concentration (copies/μL) = (6.02 × 10^23^) × (plasmid DNA concentration × 10^-9^) / (DNA length × 660). The extracted plasmid DNA is then gradient diluted for use as a standard in subsequent qPCR assays.

qPCR is performed using the Transstart® Tip Green qPCR superMix kit (TransGen Biotech, Beijing) according to the manufacturer’s instructions on the LightCycler 480 II system (Roche Diagnostics, Mannheim, Germany). The reaction system for qPCR is shown in Supplementary Table S11.

The qPCR amplification program follows the following temperature and time settings:

1) Initial denaturation: 94°C, maintained for 30 s.

2) Denaturation at 94°C (10 s), annealing (55-60°C, 15 s, optimal annealing temperature determined by primers), extension (72°C, 10 s). This step is repeated for a total of 40 cycles.

The specificity of qPCR products is verified using a melt curve analysis, with the temperature range of the melt curve varying from 65°C to 95°C.

The full-length sequences of the *Dehalococcoides* 16S rRNA gene and reductive dehalogenase genes are obtained using the following steps: PCR amplification of target genes using the above-mentioned reagents, gel extraction, transformation, and blue-white screening. Subsequently, the Escherichia coli containing the target gene fragments are sent to Shanghai Sangon Biotech Co., Ltd. for Sanger sequencing to obtain the sequences of the target gene fragments inserted into the vectors. MEGA 7.0 software is used for constructing phylogenetic trees based on the neighbor-joining method, with Bootstrap set to 1000 replicates.

**References**

Grostern, A., and Edwards, E.A. (2006). Growth of *Dehalobacter* and *Dehalococcoides* spp. during degradation of chlorinated ethanes. *Appl. Environ. Microbiol.* 72(1)**,** 428-436. doi: 10.1128/aem.72.1.428-436.2006.

Krajmalnik-Brown, R., Hölscher, T., Thomson, I.N., Saunders, F.M., Ritalahti, K.M., and Löffler, F.E. (2004). Genetic identification of a putative vinyl chloride reductase in *Dehalococcoides* sp. strain BAV1. *Appl. Environ. Microbiol.* 70(10)**,** 6347-6351. doi: 10.1128/aem.70.10.6347-6351.2004.

Mészáros, É., Imfeld, G., Nikolausz, M., and Nijenhuis, I. (2013). Occurrence of *Dehalococcoides* and reductive dehalogenase genes in microcosms, a constructed wetland and groundwater from a chlorinated ethene contaminated field site as indicators for in situ reductive dehalogenation. *Water, Air, Soil Pollut.* 224(11). doi: 10.1007/s11270-013-1768-x.

Shah, V., Wang, S., and He, J. (2013). Phylogenetically distinct bacteria involve extensive dechlorination of Aroclor 1260 in sediment-free cultures. *PLoS One* 8(3). doi: 10.1371/journal.pone.0059178.

Wang, S., Chng, K.R., Wilm, A., Zhao, S., Yang, K.-L., Nagarajan, N., et al. (2014). Genomic characterization of three unique *Dehalococcoides* that respire on persistent polychlorinated biphenyls. *Proceedings of the National Academy of Sciences* 111(33)**,** 12103-12108. doi: 10.1073/pnas.1404845111.

Watts, J.E.M., Fagervold, S.K., May, H.D., and Sowers, K.R. (2005). A PCR-based specific assay reveals a population of bacteria within the *Chloroflexi* associated with the reductive dehalogenation of polychlorinated biphenyls. *Microbiology* 151(6)**,** 2039-2046. doi: 10.1099/mic.0.27819-0.

Yan, J., Rash, B.A., Rainey, F.A., and Moe, W.M. (2009). Detection and quantification of dehalogenimonas and “*Dehalococcoides*” populations via PCR-based protocols targeting 16S rRNA genes. *Appl. Environ. Microbiol.* 75(23)**,** 7560-7564. doi: 10.1128/aem.01938-09.
